# Supplementary material for: Brain Age Prediction in Type II GM1 Gangliosidosis
Source: medRxiv. 2025 Apr 25:2025.04.23.25326206. Preprint. [Version 1] doi: 10.1101/2025.04.23.25326206 (PMC12045421; doi:10.1101/2025.04.23.25326206)
Supplement: Supplement 1 [file NIHPP2025.04.23.25326206v1-supplement-1.pdf]

medRxiv preprint doi: https://doi.org/10.1101/2025.04.23.25326206; this version posted April 25, 2025. The copyright holder for this preprint (which was not certified by peer review) is the author/funder, who has granted medRxiv a license to display the preprint in perpetuity.
This article is a US Government work. It is not subject to copyright under 17 USC 105 and is also made available for use under a CC0 license.

Brain Age Prediction in Type II GM1 Gangliosidosis

Supplementary Material

Table of Contents

Methods……………………………………………………………………………….…*28*

Supplement A: Natural History Study Participant Characteristics……………..………...………*28*

Supplement B: Neurotypical Controls Participants………………………………………………*30*

Supplement C: Linear Mixed Effects Modeling (LMEM)……...……………………………..*32*

Results……………………………………………………………………………………...*33*

Supplement D: Axial, Coronal, and Anatomical Slices of Brain Aging…………………………..*33*

Supplement E: Comparisons between Neurotypical Control Datasets…………………………..*37*

Supplement F: Linear Mixed Effects Modeling (LMEM) Outputs…………………...………..*39*

Supplement G: Linear Mixed Effects Modeling (LMEM) Estimates and Standard Errors………*41*

Supplement H: Ventral Diencephalon Predicted Brain Age……………………………………..*44*

Supplement I: Longitudinal Brain structures age gap estimation (BSAGE) ………………..…..*45*

Supplement J: Cross-Sectional Brain structures age gap estimation (BSAGE)…………………*56*

Supplement K: Comparisons Between Predicted Brain Age and Brain Volumetrics…………...*73*

Supplementary References……………………………………………………………………*83*

medRxiv preprint doi: https://doi.org/10.1101/2025.04.23.25326206; this version posted April 25, 2025. The copyright holder for this preprint (which was not certified by peer review) is the author/funder, who has granted medRxiv a license to display the preprint in perpetuity.
This article is a US Government work. It is not subject to copyright under 17 USC 105 and is also made available for use under a CC0 license.

Supplementary Methods
Supplement A: Natural History Study Participant Characteristics

Patients were enrolled in the National Human Genome Research Institute (NHGRI) study of the "Natural History of Glycosphingolipid Storage Disorders and Glycoprotein Disorders" (ClinicalTrials.gov ID: NCT00029965).[1] Demographic information for each of the 41 GM1 patients is listed in Table A1, and the age of the entire cohort is shown in Figure A1.

Table A1. Natural History Study T1-Weighted MRI Scan Age (n = 41). N/A are designated when a participant did not have the above referenced MRI scan. Specific ages were redacted per Medrxiv requirements.

Participant	Sex	GM1 Sub-type	Baseline Age (years old)	Scan #2 Age (years old)	Scan #3 Age (years old)	Scan #4 Age (years old)	Scan #5 Age (years old)	Number of T1 Scans
1	M	LI	0-5	N/A	N/A	N/A	N/A	1
2	M	LI	6-10	N/A	N/A	N/A	N/A	1
3	F	LI	0-5	N/A	N/A	N/A	N/A	1
4	F	LI	0-5	N/A	N/A	N/A	N/A	1
5	F	LI	0-5	N/A	N/A	N/A	N/A	1
6	F	LI	6-10	N/A	N/A	N/A	N/A	1
7	F	LI	0-5	N/A	N/A	N/A	N/A	1
8	M	LI	0-5	0-5	N/A	N/A	N/A	2
9	M	LI	6-10	6-10	N/A	N/A	N/A	2
10	M	LI	6-10	6-10	N/A	N/A	N/A	2
11	F	LI	0-5	0-5	N/A	N/A	N/A	2
12	F	LI	6-10	N/A	N/A	N/A	N/A	1
13	M	LI	0-5	N/A	N/A	N/A	N/A	1
14	F	LI	0-5	N/A	N/A	N/A	N/A	1
15	F	LI	0-5	0-5	N/A	N/A	N/A	2
16	M	Juv	0-5	0-5	N/A	N/A	N/A	2
17	F	Juv	0-5	0-5	6-10	6-10	6-10	5
18	F	Juv	6-10	N/A	N/A	N/A	N/A	1
19	F	Juv	0-5	N/A	N/A	N/A	N/A	1
20	M	Juv	6-10	N/A	N/A	N/A	N/A	1
21	M	Juv	6-10	N/A	N/A	N/A	N/A	1
22	F	Juv	6-10	N/A	N/A	N/A	N/A	1
23	M	Juv	0-5	N/A	N/A	N/A	N/A	1
24	M	Juv	0-5	N/A	N/A	N/A	N/A	1
25	M	Juv	16-20	21-25	21-25	21-25	N/A	4
26	M	Juv	11-15	11-15	16-20	16-20	21-25	5
27	F	Juv	11-15	11-15	16-20	21-25	N/A	4
28	M	Juv	11-15	11-15	16-20	16-20	N/A	4
29	F	Juv	11-15	11-15	11-15	N/A	N/A	3
30	F	Juv	11-15	11-15	16-20	N/A	N/A	3
31	M	Juv	11-15	11-15	16-20	N/A	N/A	3
32	M	Juv	6-10	11-15	N/A	N/A	N/A	2
33	F	Juv	16-20	16-20	21-25	N/A	N/A	3
34	F	Juv	6-10	6-10	6-10	N/A	N/A	3
35	F	Juv	11-15	16-20	16-20	N/A	N/A	3
36	F	Juv	6-10	6-10	N/A	N/A	N/A	2
37	M	Juv	6-10	6-10	N/A	N/A	N/A	2
38	F	Juv	6-10	11-15	N/A	N/A	N/A	2
39	F	Juv	11-15	N/A	N/A	N/A	N/A	1

medRxiv preprint doi: https://doi.org/10.1101/2025.04.23.25326206; this version posted April 25, 2025. The copyright holder for this preprint (which was not certified by peer review) is the author/funder, who has granted medRxiv a license to display the preprint in perpetuity.
This article is a US Government work. It is not subject to copyright under 17 USC 105 and is also made available for use under a CC0 license.

40	F	Juv	6-10	11-15	N/A	N/A	N/A	2
41	F	Juv	0-5	N/A	N/A	N/A	N/A	1

medRxiv preprint doi: https://doi.org/10.1101/2025.04.23.25326206; this version posted April 25, 2025. The copyright holder for this preprint (which was not certified by peer review) is the author/funder, who has granted medRxiv a license to display the preprint in perpetuity. This article is a US Government work. It is not subject to copyright under 17 USC 105 and is also made available for use under a CC0 license.

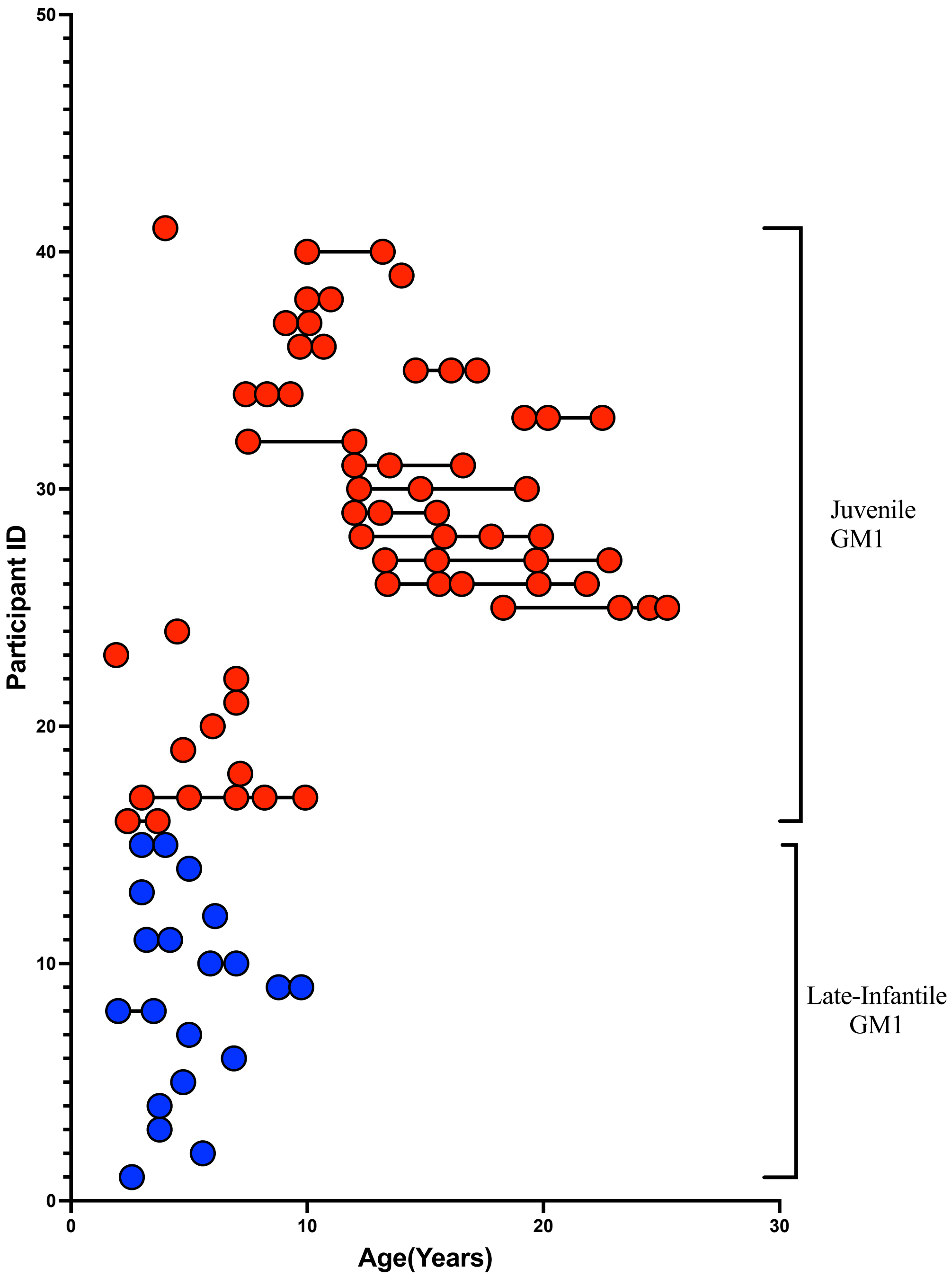

Figure A1. GM1 patient age at each T1-weighted MRI Scan. Late-Infantile (LI, n=15, 20 total MRI scans) GM1 patients are shown in blue and juvenile (n = 26, 61 total MRI scans) GM1 patients are red. Each T1-weighted scan is represented as a circle for all 65 scans; connecting lines indicate that a repeated scan was collected on the same patient. Each patient is shown on a separate row.

medRxiv preprint doi: https://doi.org/10.1101/2025.04.23.25326206; this version posted April 25, 2025. The copyright holder for this preprint (which was not certified by peer review) is the author/funder, who has granted medRxiv a license to display the preprint in perpetuity. This article is a US Government work. It is not subject to copyright under 17 USC 105 and is also made available for use under a CC0 license.

Supplement B: Neurotypical Control Participants

Table BI. Summary of Neurotypical Control Data Sets

	Calgary[2,3]	QTAB[4,5]	QTIM[6,7]
**n**	97	159	300
**Baseline Age ($\bar{x}$ ± SD)**	3.84 ± 0.88	10.93 ± 1.36	21.72 ± 3.71
**Age (min, max)**	(1.97, 6.9)	(8, 16)	(12, 30)
**Males (n)**	51	82	123
**Females (n)**	46	77	177

First, 97 participants (279 scans) from the Calgary Preschool MRI dataset were included consisting of participants between the ages of 2 and 7 years old.[2,3] Second, 159 participants (318 scans) from the Queensland Twin Adolescent Brain (QTAB) were included in this study, consisting of participants between the ages of 8 and 16 years old.[4,5] Lastly, 300 participants (300 scans) from the Queensland Twin IMaging (QTIM) were included in this study, consisting of participants between the ages of 12 and 30.[6,7]

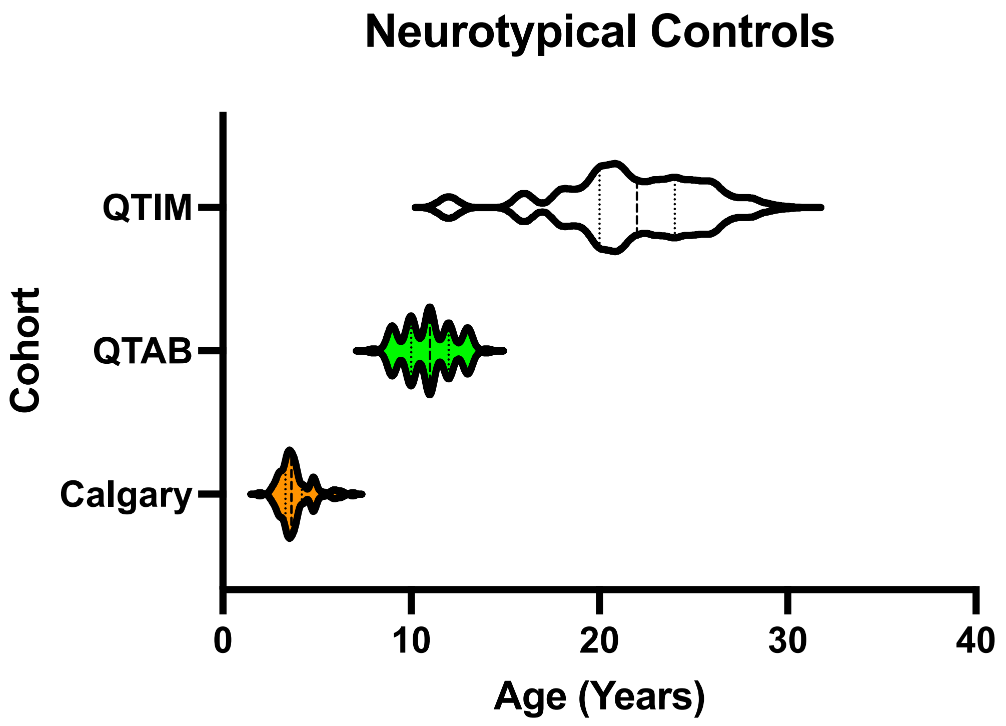

Figure B1. Violin Plot of Neurotypical Control Age. Baseline Scans were used for the Calgary and QTAB Datasets.

medRxiv preprint doi: https://doi.org/10.1101/2025.04.23.25326206; this version posted April 25, 2025. The copyright holder for this preprint (which was not certified by peer review) is the author/funder, who has granted medRxiv a license to display the preprint in perpetuity. This article is a US Government work. It is not subject to copyright under 17 USC 105 and is also made available for use under a CC0 license.

Supplement C: Linear Mixed Effects Modeling

Testing the interaction of Cohort and Age on predicted brain age
Linear mixed effects modeling was built in R[8] with the LME4[9-11] package. The interaction between the cohort and biological age was tested using the sample code below:

Model 1:
```
model1 <- lmer(Predicted Brain Age ~ Age * Cohort + Sex + (1|Participant), dataset)
summary(lmer)
```

Model 2:
```
model2 <- lmer(Predicted Brain Age ~ Age + Cohort + Sex + (1|Participant), dataset)
summary(lmer)
```

Likelihood-ratio test:
```
fm.anova <- anova(model1,model2)
summary(fm.anova)
```

Predicted Brain Age – Corresponds to the predicted brain age determined by *BrainStructuresAges* for the structure being analyzed
Cohort – Corresponds to the comparison between cohorts as presented in Table 1.
Sex – Corresponds to the participants' biological sex
Age – Corresponds to the participants' chronological age
Participant – Each participant was given a distinct number to account for repeated measures (subject level random intercept).

medRxiv preprint doi: https://doi.org/10.1101/2025.04.23.25326206; this version posted April 25, 2025. The copyright holder for this preprint (which was not certified by peer review) is the author/funder, who has granted medRxiv a license to display the preprint in perpetuity.
This article is a US Government work. It is not subject to copyright under 17 USC 105 and is also made available for use under a CC0 license.

Supplementary Results

Supplement D: Axial, Coronal, and Anatomical Slices of Brain Aging

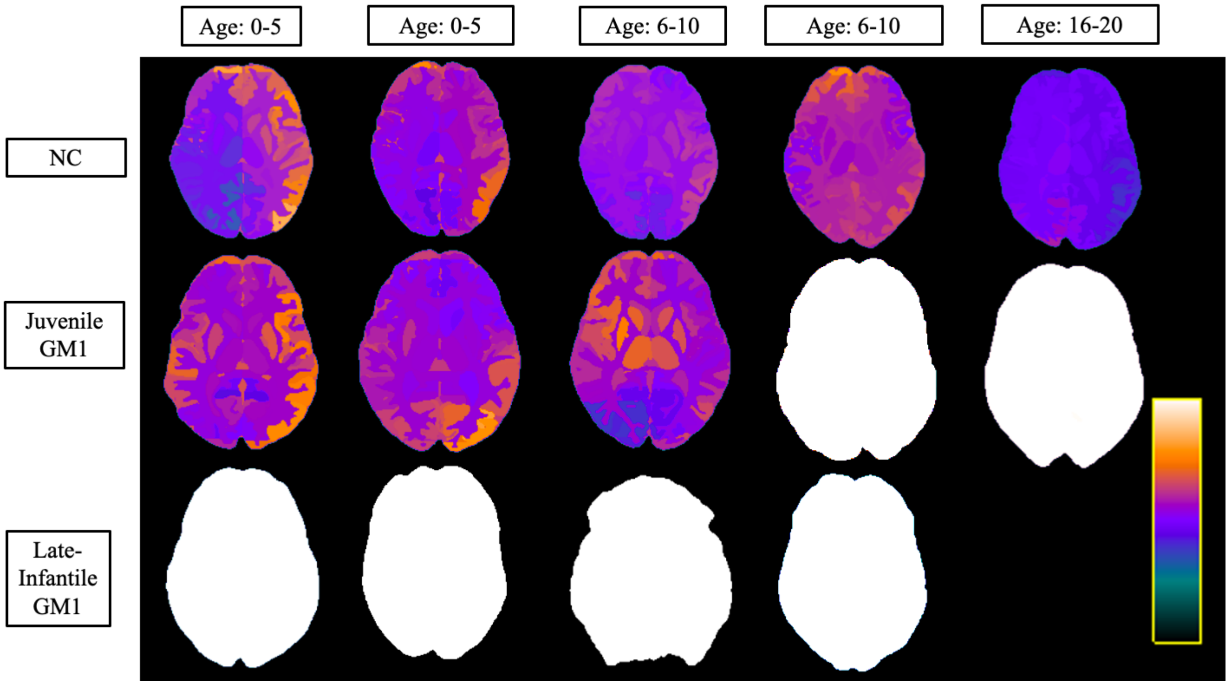

Figure D1. Axial View of Brain Structures Ages of the Three Cohorts at Varying Ages. Neurotypical controls (NC) are shown on the top row, juvenile GM1 gangliosidosis patients are shown in the middle row, and late-infantile GM1 gangliosidosis patients are shown in the bottom row. Colors were assigned according to the predicted age of that brain structure and were assigned between very dark blue (lowest predicted age) and white (highest predicted age) as shown in the scalar bar in the bottom right portion. The scalar ranges were adjusted for each column with min/max values of 0-8 years for the first column (0-5 years old), 0-10 years for the second column (0-5 years old), 0-14 years for the third column (6-10 years old), 0-20 years for the fourth column (6-10 years old), and 0-40 years for the fifth column (16-20 years old). No 20-year-old MRI scan was available for late-infantile patients as the oldest late-infantile patient in this study was 6-10 years old at the time of the MRI evaluation. All MRI images were registered to the MNI coordinate space and slices were taken at z = 6.4 (± 1) mm for each participant. A matching anatomical version of this image is available (Supplement Figure D4). Specific ages were redacted per Medrxiv requirements.

medRxiv preprint doi: https://doi.org/10.1101/2025.04.23.25326206; this version posted April 25, 2025. The copyright holder for this preprint
(which was not certified by peer review) is the author/funder, who has granted medRxiv a license to display the preprint in perpetuity.
This article is a US Government work. It is not subject to copyright under 17 USC 105 and is also made available
for use under a CC0 license.

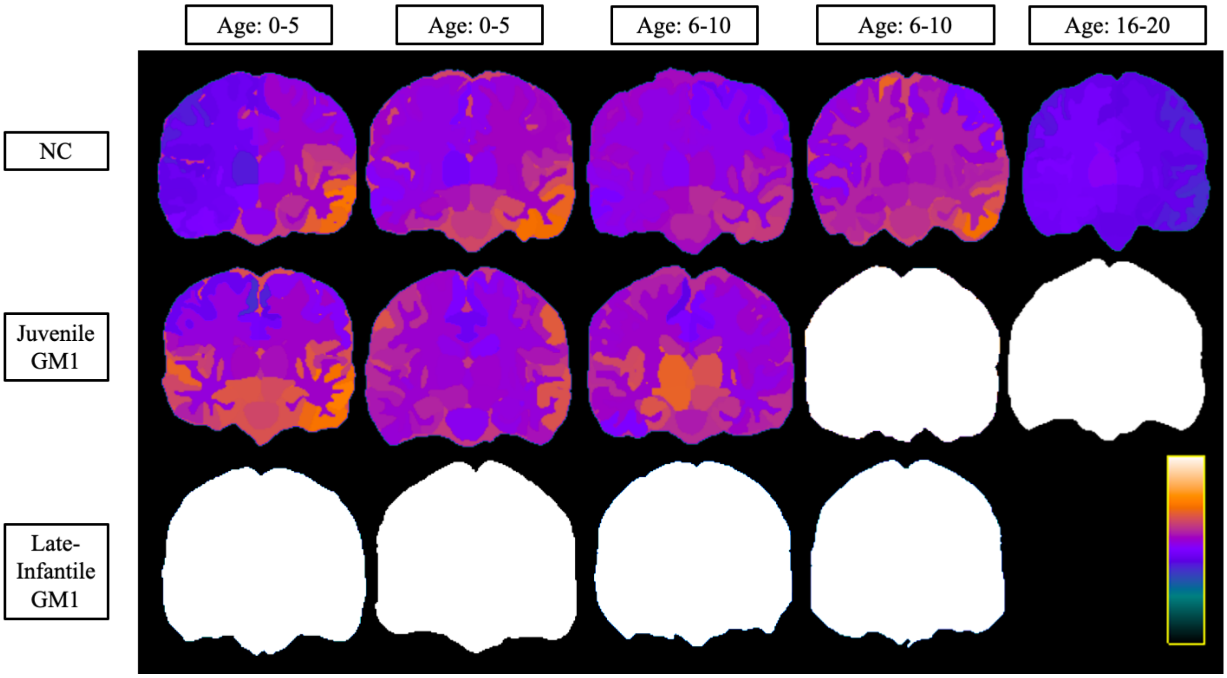

Figure D2. Coronal View of Brain Structures Ages of the Three Cohorts at Varying Ages. Neurotypical controls (NC) are shown on the top row, juvenile GM1 gangliosidosis patients are shown in the middle row, and late-infantile GM1 gangliosidosis patients are shown in the bottom row. Colors were assigned according to the predicted age of that brain structure and were assigned between very dark blue (lowest predicted age) and white (highest predicted age) as shown in the scalar bar in the bottom right portion. The scalar ranges were adjusted for each column with min/max values of 0-8 years for the first column (0-5 years old), 0-10 years for the second column (0-5 years old), 0-14 years for the third column (6-10 years old), 0-20 years for the fourth column (6-10 years old), and 0-40 years for the fifth column (16-20 years old). No 20-year-old MRI scan was available for late-infantile patients as the oldest late-infantile patient in this study was 6-10 years old at the time of the MRI evaluation. All MRI images were registered to the MNI coordinate space and slices were taken at y = -17.6 (± 1) mm for each participant. A matching anatomical version of this image is available (Supplement Figure D5). Specific ages were redacted per Medrxiv requirements.

medRxiv preprint doi: https://doi.org/10.1101/2025.04.23.25326206; this version posted April 25, 2025. The copyright holder for this preprint (which was not certified by peer review) is the author/funder, who has granted medRxiv a license to display the preprint in perpetuity.
This article is a US Government work. It is not subject to copyright under 17 USC 105 and is also made available for use under a CC0 license.

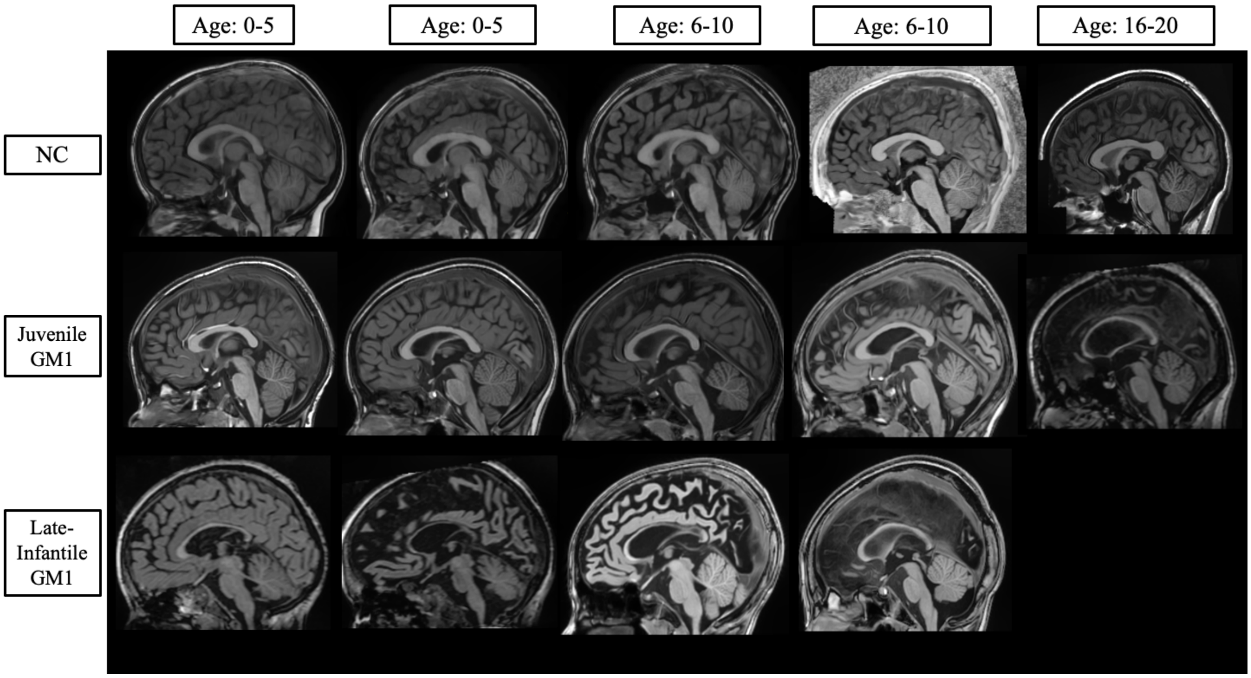

Figure D3. T1-Weighted Sagittal Anatomical Slice of Figure 1. Neurotypical controls (NC) are shown on the top row, juvenile GM1 gangliosidosis patients are shown in the middle row, and late-infantile GM1 gangliosidosis patients are shown in the bottom row. All MRI images were registered to the MNI coordinate space and slices were taken at x = 1.0 (± 1) mm (identical to Figure 2) for each participant. Specific ages were redacted per Medrxiv requirements.

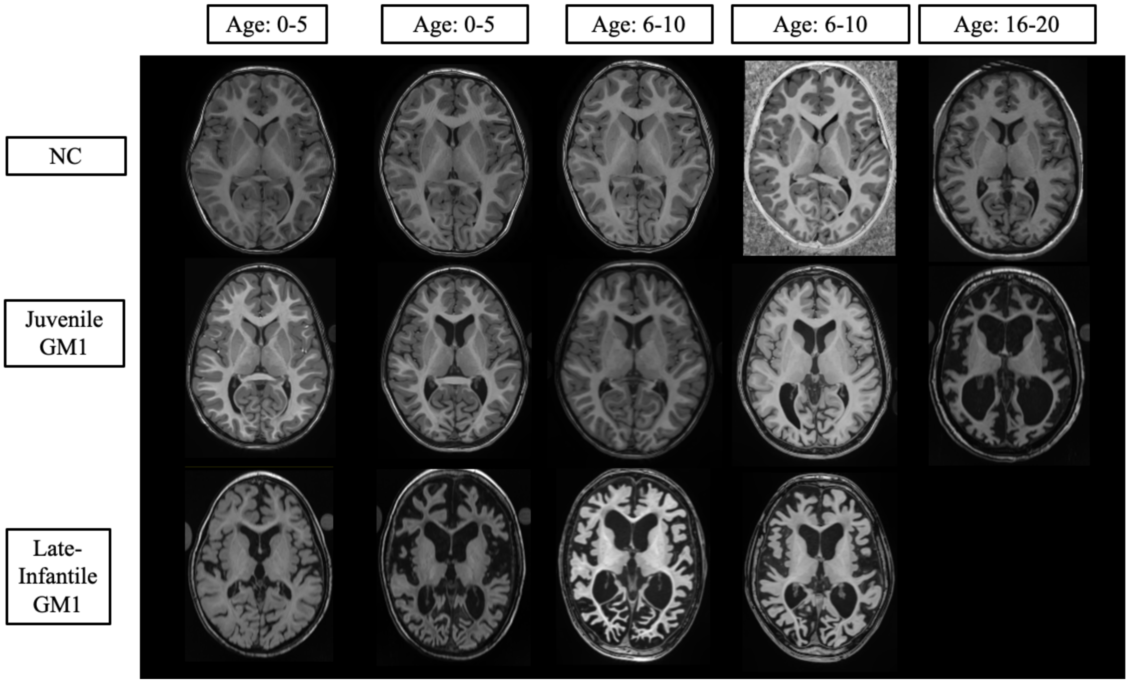

Figure D4. T1-Weighted Axial Anatomical Slice of Figure D1. Neurotypical controls (NC) are shown on the top row, juvenile GM1 gangliosidosis patients are shown in the middle row, and

medRxiv preprint doi: https://doi.org/10.1101/2025.04.23.25326206; this version posted April 25, 2025. The copyright holder for this preprint (which was not certified by peer review) is the author/funder, who has granted medRxiv a license to display the preprint in perpetuity. This article is a US Government work. It is not subject to copyright under 17 USC 105 and is also made available for use under a CC0 license.

late-infantile GM1 gangliosidosis patients are shown in the bottom row. All MRI images were registered to the MNI coordinate space and slices were taken at z = 6.4 (± 1) mm (identical to Figure D1) for each participant. Specific ages were redacted per Medrxiv requirements.

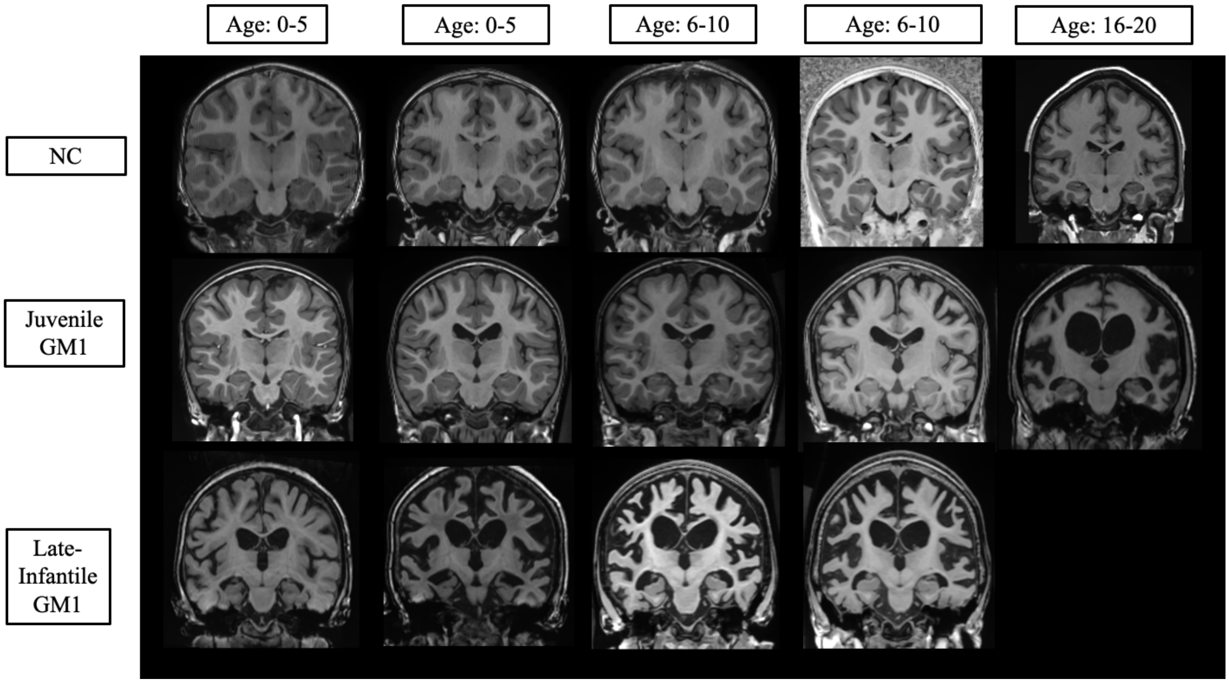

Figure D5. T1-Weighted Coronal Anatomical Slice of Figure D2. Neurotypical controls (NC) are shown on the top row, juvenile GM1 gangliosidosis patients are shown in the middle row, and late-infantile GM1 gangliosidosis patients are shown in the bottom row. All MRI images were registered to the MNI coordinate space and slices were taken at y = -17.6 (± 1) mm (identical to Figure D2) for each participant. Specific ages were redacted per Medrxiv requirements.

medRxiv preprint doi: https://doi.org/10.1101/2025.04.23.25326206; this version posted April 25, 2025. The copyright holder for this preprint (which was not certified by peer review) is the author/funder, who has granted medRxiv a license to display the preprint in perpetuity. This article is a US Government work. It is not subject to copyright under 17 USC 105 and is also made available for use under a CC0 license.

Supplement E: Comparisons between Neurotypical Control Datasets

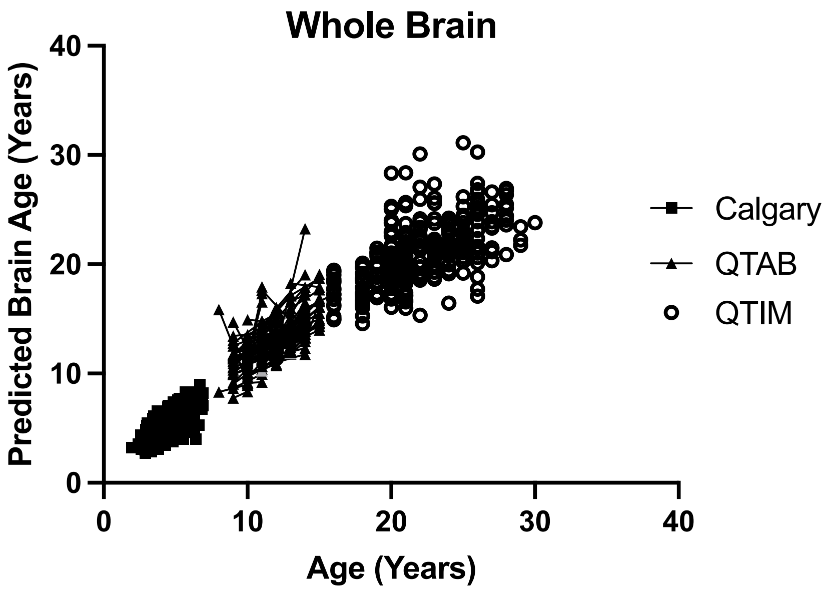

Figure E1. The relationship between predicted brain age and biological age in NC participants (n = 556, 897 total MRI scans). Calgary participants are shown as squares, QTAB participants are shown as triangles, and QTIM participants are shown as open circles. Connecting lines indicate repeated scans on the same participant. Calgary participants showed an average increase of 0.77 (0.03) in predicted brain age per biological year, QTAB participants showed an average increase of 0.81 (0.05) in predicted brain age per biological year, and QTIM participants showed an average increase of 0.65 (0.03) in predicted brain age per biological year

medRxiv preprint doi: https://doi.org/10.1101/2025.04.23.25326206; this version posted April 25, 2025. The copyright holder for this preprint (which was not certified by peer review) is the author/funder, who has granted medRxiv a license to display the preprint in perpetuity. This article is a US Government work. It is not subject to copyright under 17 USC 105 and is also made available for use under a CC0 license.

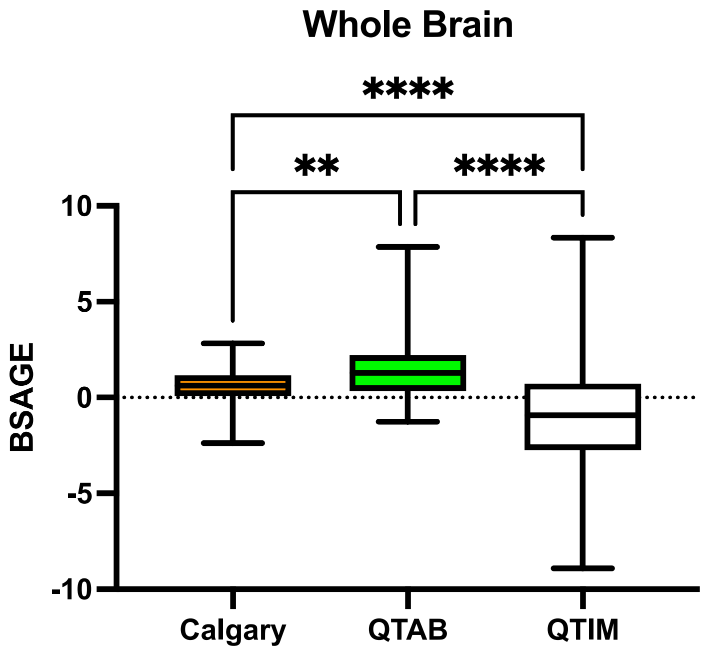

Figure E2. Comparison of Whole Brain BSAGE between NC participants (n = 556). Calgary participants had an average BSAGE = 0.63, QTAB participants had an average BSAGE = 1.48, and QTIM participants had an average BSAGE = -0.93. An analysis of variance (ANOVA) showed statistically significant ($F(2, 554) = 43.53$, $p < 0.0001$) differences in BSAGE between the three NC cohorts. A post-hoc Tukey test showed the QTAB cohort had a statistically significantly higher BSAGE compared to both Calgary ($p = 0.009$) and QTIM ($p < 0.0001$). The Calgary cohort also had a significantly higher BSAGE compared to QTIM ($p < 0.0001$).

medRxiv preprint doi: https://doi.org/10.1101/2025.04.23.25326206; this version posted April 25, 2025. The copyright holder for this preprint (which was not certified by peer review) is the author/funder, who has granted medRxiv a license to display the preprint in perpetuity.
This article is a US Government work. It is not subject to copyright under 17 USC 105 and is also made available for use under a CC0 license.

Supplement F: Linear Mixed Effects Modeling (LMEM) Outputs

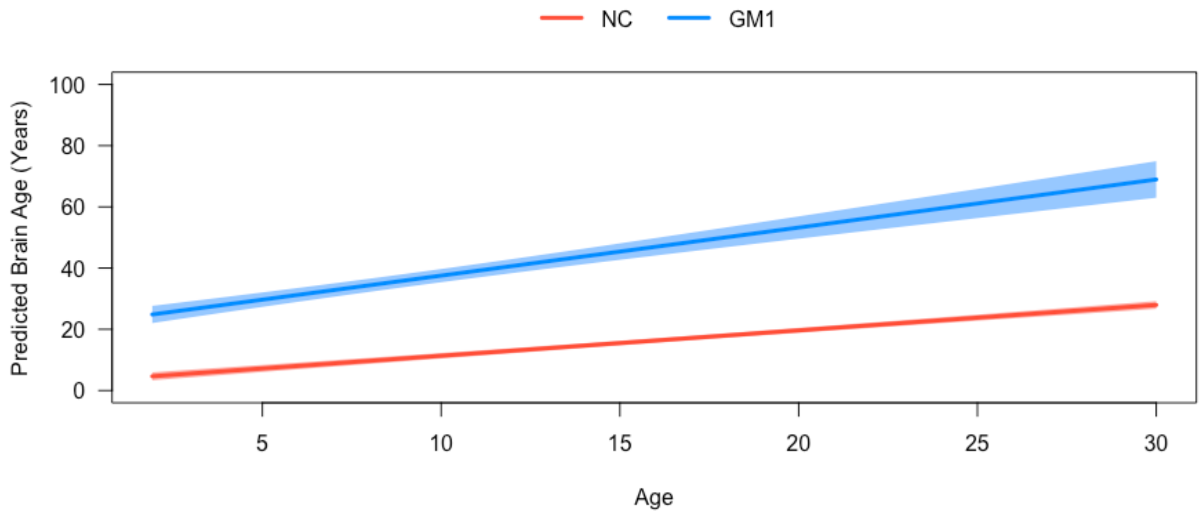

Figure F1. Comparisons of GM1 patients and neurotypical controls (NC) for predicted whole brain age as assessed by LMEM. The GM1 cohort is shown in blue, and the neurotypical controls are shown in red. The shaded regions represent the 95% confidence intervals calculated from the estimates and standards errors defined in Table G1.

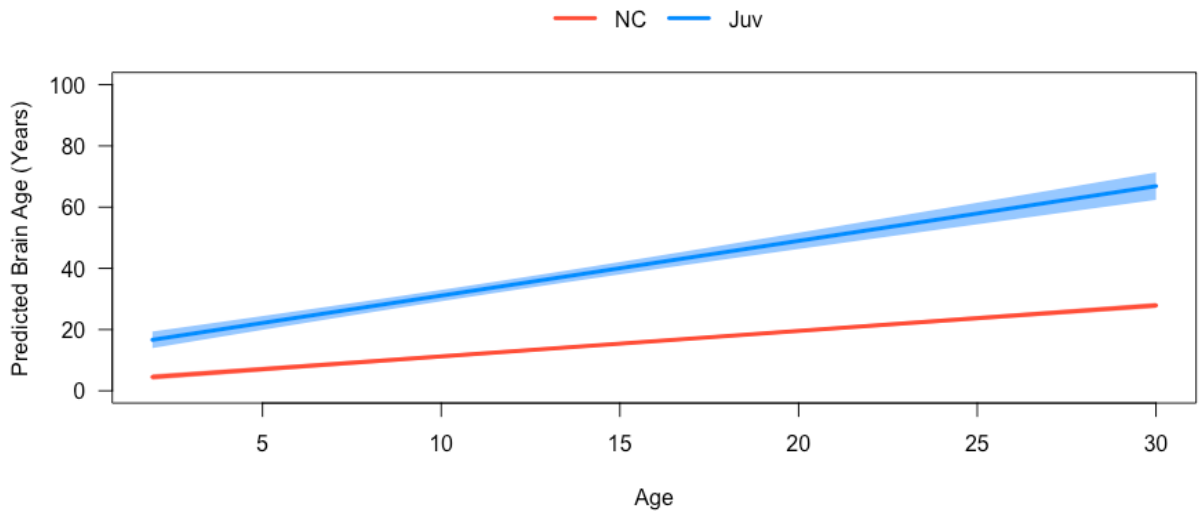

Figure F2. Comparisons of juvenile (Juv) GM1 patients and neurotypical controls (NC) for predicted whole brain age as assessed by LMEM. The Juv GM1 cohort is shown in blue, and the NC are shown in red. The shaded regions represent the 95% confidence intervals calculated from the estimates and standards errors defined in Table G1.

medRxiv preprint doi: https://doi.org/10.1101/2025.04.23.25326206; this version posted April 25, 2025. The copyright holder for this preprint (which was not certified by peer review) is the author/funder, who has granted medRxiv a license to display the preprint in perpetuity. This article is a US Government work. It is not subject to copyright under 17 USC 105 and is also made available for use under a CC0 license.

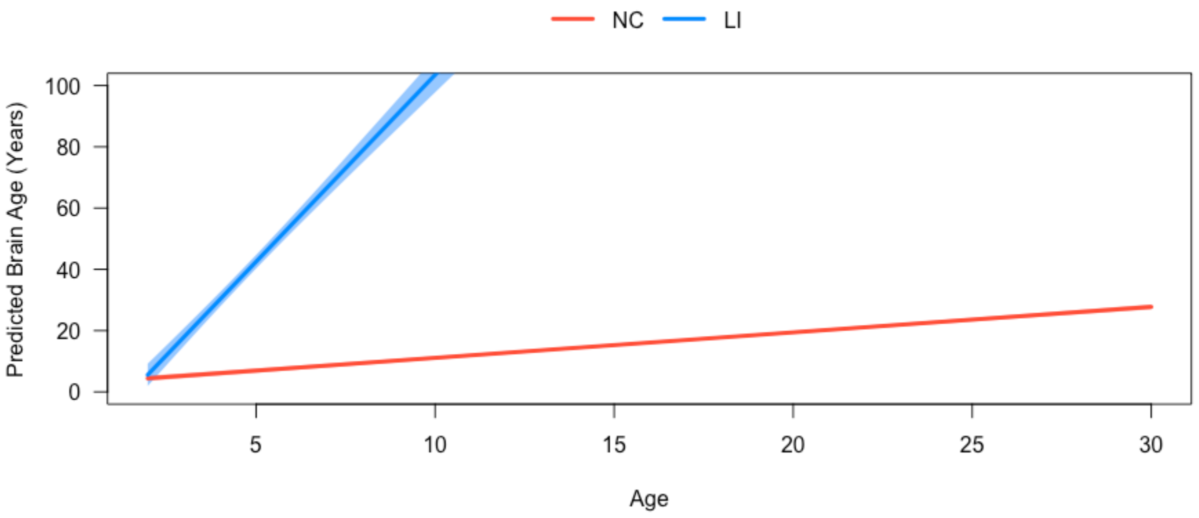

Figure F3. Comparisons of late-infantile (LI) GM1 patients and neurotypical controls for predicted whole brain age as assessed by LMEM. The LI GM1 cohort is shown in blue, and the neurotypical controls are shown in red. The shaded regions represent the 95% confidence intervals calculated from the estimates and standards errors defined in Table G1.

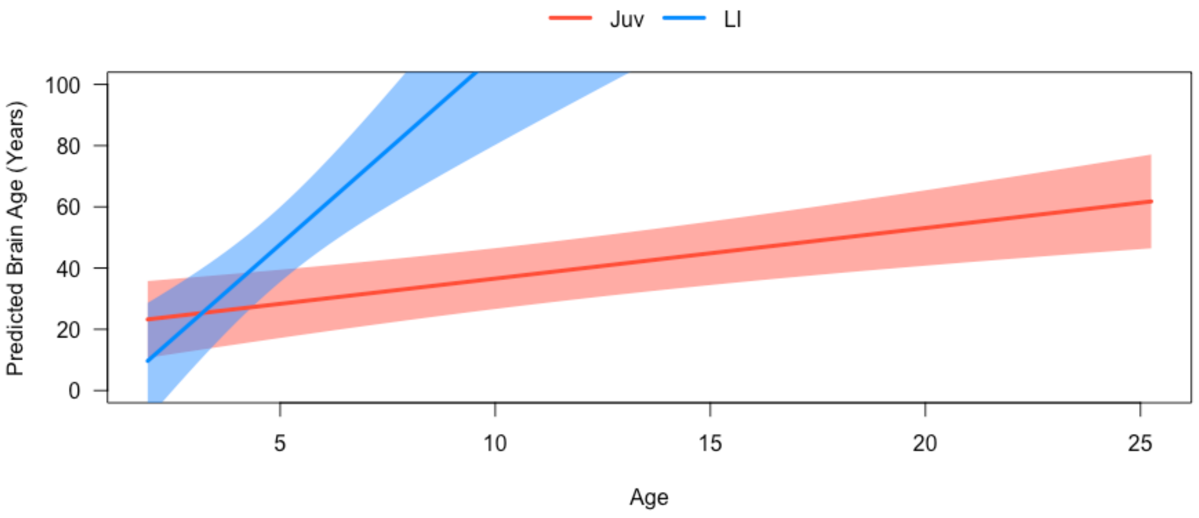

Figure F4. Comparisons of late-infantile (LI) GM1 patients and Juvenile (Juv) GM1 patients for predicted whole brain age as assessed by LMEM. The LI GM1 cohort is shown in blue, and the Juv GM1 patients are shown in red. The shaded regions represent the 95% confidence intervals calculated from the estimates and standards errors defined in Table G1.

medRxiv preprint doi: https://doi.org/10.1101/2025.04.23.25326206; this version posted April 25, 2025. The copyright holder for this preprint (which was not certified by peer review) is the author/funder, who has granted medRxiv a license to display the preprint in perpetuity. This article is a US Government work. It is not subject to copyright under 17 USC 105 and is also made available for use under a CC0 license.

Supplement G: Linear Mixed Effects Modeling (LMEM) Estimates and Standard Errors

Supplement Table G1. Estimates and Standard Errors from the LMEM for comparisons of the whole GM1 cohort, Late-Infantile (LI) GM1 patients, and Juvenile (Juv) GM1 patients, and Neurotypical controls (NC) for each of the structures analyzed. The estimates for the intercept, followed by the interaction between the cohort and biological age, biological age alone, the cohort alone, and biological sex are defined in each cell with the standard errors in parenthesis.

Structure	*GM1 v NC*	*Juv v NC*	*LI v NC*	*Juv v LI*
Intercept: Estimate (Standard Error)<br>Interaction: Estimate (Standard Error)<br>Age: Estimate (Standard Error)<br>Cohort: Estimate (Standard Error)<br>Sex: Estimate (Standard Error)				
**Whole Brain**	3.06 (0.75)<br>0.74 (0.14)<br>0.83 (0.04)<br>18.77 (1.73)<br>-0.74 (0.57)	2.90 (0.53)<br>0.96 (0.11)<br>0.83 (0.03)<br>10.31 (1.61)<br>-0.51 (0.40)	2.79 (0.47)<br>11.37 (0.52)<br>0.83 (0.02)<br>-21.29 (2.76)<br>-0.24 (0.36)	20.06 (6.93)<br>10.70 (2.63)<br>1.65 (0.42)<br>-34.12 (15.04)<br>-13.06 (6.77)
**White Matter**	3.76 (0.76)<br>0.92 (0.14)<br>0.77 (0.04)<br>18.94 (1.77)<br>-0.76 (0.57)	3.59 (0.57)<br>1.09 (0.12)<br>0.78 (0.03)<br>12.02 (1.74)<br>-0.47 (0.43)	3.45 (0.49)<br>10.57 (0.54)<br>0.78 (0.03)<br>-18.32 (2.87)<br>-0.23 (0.38)	22.81 (7.27)<br>9.67 (2.76)<br>1.73 (0.45)<br>-32.39 (15.78)<br>-13.92 (7.03)
**3rd Ventricle**	3.74 (0.77)<br>0.62 (0.13)<br>0.79 (0.04)<br>20.38 (1.70)<br>-0.79 (0.58)	3.50 (0.53)<br>0.83 (0.11)<br>0.80 (0.03)<br>12.35 (1.57)<br>-0.40 (0.41)	3.41 (0.49)<br>8.98 (0.53)<br>0.80 (0.02)<br>-9.51 (2.84)<br>-0.22 (0.38)	23.85 (6.81)<br>8.48 (2.58)<br>1.49 (0.40)<br>-24.57 (14.82)<br>-15.87 (6.87)
**4th Ventricle**	5.04 (0.74)<br>0.42 (0.11)<br>0.74 (0.04)<br>17.31 (1.58)<br>-0.73 (0.57)	4.88 (0.51)<br>0.47 (0.08)<br>0.74 (0.03)<br>9.65 (1.33)<br>-0.46 (0.39)	4.76 (0.45)<br>11.30 (0.49)<br>0.74 (0.02)<br>-22.22 (2.61)<br>-0.23 (0.35)	20.68 (5.69)<br>11.40 (2.02)<br>1.15 (0.28)<br>-35.25 (12.03)<br>-13.43 (6.42)
**Inferior Lateral Ventricles**	3.30 (0.76)<br>0.42 (0.11)<br>0.81 (0.04)<br>22.58 (1.61)<br>-0.85 (0.58)	3.10 (0.56)<br>0.50 (0.10)<br>0.82 (0.03)<br>16.69 (1.53)<br>-0.54 (0.43)	2.99 (0.48)<br>8.60 (0.45)<br>0.82 (0.02)<br>-7.93 (2.50)<br>-0.25 (0.38)	27.48 (6.47)<br>8.45 (2.32)<br>1.19 (0.32)<br>-27.43 (13.76)<br>-15.32 (7.22)
**Lateral Ventricles**	5.04 (0.76)<br>0.94 (0.15)<br>0.70 (0.04)<br>17.72 (1.78)<br>-0.70 (0.57)	4.87 (0.55)<br>1.16 (0.12)<br>0.71 (0.03)<br>9.81 (1.71)<br>-0.40 (0.42)	4.69 (0.50)<br>10.40 (0.54)<br>0.71 (0.03)<br>-17.86 (2.91)<br>-0.16 (0.38)	21.95 (7.35)<br>9.36 (2.78)<br>1.74 (0.46)<br>-29.34 (15.90)<br>-14.18 (2.78)
**Ventral Diencephalon**	4.19 (0.75)<br>0.58 (0.13)<br>0.77 (0.04)<br>19.89 (1.68)<br>-0.75 (0.57)	4.19 (0.75)<br>0.70 (0.10)<br>0.77 (0.04)<br>19.89 (1.68)<br>-0.75 (0.57)	3.87 (0.46)<br>11.54 (0.50)<br>0.77 (0.02)<br>-21.35 (2.69)<br>-0.21 (0.36)	23.46 (6.46)<br>11.36 (2.43)<br>1.36 (0.37)<br>-37.08 (14.04)<br>-14.62 (6.73)
**External CSF**	5.14 (0.74)<br>1.04 (0.14)<br>0.73 (0.04)<br>16.45 (1.73)<br>-0.80 (0.56)	5.14 (0.74)<br>1.20 (0.12)<br>0.73 (0.04)<br>16.45 (1.73)<br>-0.80 (0.56)	4.85 (0.47)<br>9.76 (0.53)<br>0.74 (0.02)<br>-16.91 (2.80)<br>-0.30 (0.36)	21.58 (7.03)<br>8.83 (2.67)<br>1.83 (0.44)<br>-29.15 (15.23)<br>-13.29 (6.74)
**Thalamus**	3.34 (0.76)	3.34 (0.76)	3.03 (0.50)	23.50 (6.94)

medRxiv preprint doi: https://doi.org/10.1101/2025.04.23.25326206; this version posted April 25, 2025. The copyright holder for this preprint (which was not certified by peer review) is the author/funder, who has granted medRxiv a license to display the preprint in perpetuity.
This article is a US Government work. It is not subject to copyright under 17 USC 105 and is also made available for use under a CC0 license.

	0.63 (0.13)	0.81 (0.11)	10.57 (0.52)	9.94 (2.62)
	0.80 (0.04)	0.80 (0.04)	0.80 (0.03)	1.47 (0.40)
	20.65 (1.72)	20.65 (1.72)	-16.54 (2.82)	-31.39 (15.09)
	-0.75 (0.58)	-0.75 (0.58)	-0.19 (0.39)	-14.55 (7.09)
**Caudate Nucleus**	3.44 (0.78)	3.44 (0.78)	3.13 (0.53)	21.17 (8.01)
	1.05 (0.15)	1.16 (0.13)	9.30 (0.60)	8.57 (3.03)
	0.79 (0.04)	0.79 (0.04)	0.80 (0.03)	1.80 (0.51)
	15.88 (1.84)	15.88 (1.84)	-17.63 (3.18)	-32.17 (17.30)
	-0.64 (0.59)	-0.64 (0.59)	-0.07 (0.41)	-11.83 (7.58)
**Nucleus Accumbens**	3.82 (0.73)	3.82 (0.73)	3.50 (0.49)	21.01 (8.35)
	0.93 (0.15)	0.98 (0.14)	7.37 (0.59)	6.68 (3.10)
	0.79 (0.04)	0.79 (0.04)	0.79 (0.03)	1.65 (0.56)
	14.63 (1.82)	14.63 (1.82)	-12.17 (3.11)	-26.48 (17.68)
	-0.42 (0.56)	-0.42 (0.56)	0.14 (0.38)	-9.81 (7.29)
**Putamen**	4.01 (0.78)	3.86 (0.55)	3.69 (0.50)	25.19 (7.38)
	0.82 (0.14)	0.97 (0.12)	11.30 (0.54)	10.56 (2.80)
	0.76 (0.04)	0.76 (0.03)	0.76 (0.03)	1.55 (0.44)
	20.21 (1.78)	13.46 (1.68)	-21.02 (2.90)	-36.99 (16.06)
	-0.71 (0.59)	-0.47 (0.42)	-0.12 (0.39)	-14.31 (7.39)
**Globus Pallidus**	3.82 (0.78)	3.66 (0.55)	3.52 (0.51)	24.30 (7.42)
	0.77 (0.14)	0.93 (0.42)	11.11 (0.55)	10.33 (2.81)
	0.78 (0.04)	0.78 (0.03)	0.78 (0.03)	1.54 (0.44)
	19.97 (1.78)	13.04 (1.66)	-20.39 (2.96)	-35.49 (16.14)
	-0.70 (0.59)	-0.47 (0.42)	-0.12 (0.40)	-13.98 (7.43)
**Hippocampus**	4.54 (0.74)	4.41 (0.53)	4.29 (0.47)	26.36 (6.61)
	0.51 (0.12)	0.56 (0.10)	10.85 (0.49)	10.67 (2.37)
	0.73 (0.04)	0.74 (0.03)	0.74 (0.02)	1.22 (0.34)
	21.92 (1.66)	16.08 (1.56)	-19.08 (2,64)	-37.20 (14.07)
	-0.52 (0.57)	-0.28 (0.41)	-0.12 (0.37)	-13.55 (7.45)
**Basal Forebrain**	4.63 (0.78)	4.49 (0.56)	4.30 (0.49)	23.93 (7.36)
	0.84 (0.15)	0.98 (0.12)	12.04 (0.56)	11.63 (2.79
	0.76 (0.04)	0.76 (0.03)	0.76 (0.03)	1.60 (0.45)
	19.11 (1.82)	12.09 (1.71)	-24.73 (2.95)	-40.74 (15.97)
	-0.62 (0.59)	-0.39 (0.43)	-0.05 (0.38)	-14.00 (7.17)
**Amygdala**	4.70 (0.78)	4.70 (0.78)	4.34 (0.49)	25.70 (6.83)
	0.68 (0.13)	0.77 (0.11)	10.50 (0.51)	10.07 (2.53)
	0.76 (0.04)	0.76 (0.04)	0.76 (0.03)	1.37 (0.37)
	19.48 (1.70)	19.48 (1.70)	-18.77 (2.77)	-35.18 (14.77)
	-0.66 (0.59)	-0.66 (0.59)	-0.06 (0.38)	-14.47 (7.32)
**Cerebellar Gray Matter**	6.36 (0.71)	6.36 (0.71)	6.09 (0.45)	21.80 (5.60)
	0.38 (0.11)	0.44 (0.08)	10.61 (0.49)	10.89 (2.05)
	0.65 (0.04)	0.65 (0.04)	0.65 (0.02)	1.04 (0.29)
	16.61 (1.55)	16.61 (1.55)	-20.90 (2.63)	-34.45 (12.02)
	-0.72 (0.54)	-0.72 (0.54)	-0.20 (0.35)	-13.46 (6.11)
**Cerebellar White Matter**	5.55 (0.73)	5.55 (0.73)	5.26 (0.45)	21.66 (5.80)
	0.43 (0.12)	0.46 (0.08)	11.04 (0.50)	11.32 (2.10)
	0.70 (0.04)	0.70 (0.04)	0.70 (0.02)	1.12 (0.29)
	17.33 (1.59)	17.33 (1.59)	-21.60 (2.65)	-35.91 (12.38)
	-0.77 (0.56)	-0.77 (0.56)	-0.23 (0.35)	-13.77 (6.43)
**Cerebellar Lobule I-V**	4.42 (0.76)	4.42 (0.76)	4.09 (0.47)	25.40 (6.49)
	0.56 (0.13)	0.64 (0.10)	11.22 (0.49)	10.93 (2.41)
	0.76 (0.04)	0.76 (0.04)	0.76 (0.02)	1.30 (0.35)
	20.54 (1.67)	20.54 (1.67)	-20.07 (2.67)	-36.45 (14.02)
	-0.80 (0.58)	-0.80 (0.58)	-0.23 (0.37)	-15.26 (6.94)
**Cerebellar Lobule VI-VII**	5.13 (0.71)	5.13 (0.71)	4.86 (0.46)	20.95 (5.68)
	0.45 (0.11)	0.52 (0.08)	10.46 (0.50)	10.52 (2.09)
	0.72 (0.04)	0.72 (0.04)	0.73 (0.02)	1.21 (0.30)
	17.44 (1.55)	17.44 (1.55)	-18.56 (2.67)	-31.90 (12.23)
	-0.70 (0.54)	-0.70 (0.54)	-0.23 (0.35)	-13.56 (6.15)
**Cerebellar Lobule VIII-X**	5.24 (0.70)	5.24 (0.70)	4.97 (0.45)	20.94 (5.58)
	0.38 (0.11)	0.44 (0.08)	9.57 (0.47)	9.42 (2.02)

medRxiv preprint doi: https://doi.org/10.1101/2025.04.23.25326206; this version posted April 25, 2025. The copyright holder for this preprint (which was not certified by peer review) is the author/funder, who has granted medRxiv a license to display the preprint in perpetuity.
This article is a US Government work. It is not subject to copyright under 17 USC 105 and is also made available for use under a CC0 license.

	0.72 (0.04)<br>17.00 (1.50)<br>-0.73 (0.54)	0.72 (0.04)<br>17.00 (1.50)<br>-0.73 (0.54)	0.72 (0.02)<br>-15.69 (2.54)<br>-0.23 (0.35)	1.12 (0.28)<br>-27.54 (11.92)<br>-13.40 (6.18)
**Brainstem**	4.80 (0.75)<br>0.51 (0.13)<br>0.75 (0.04)<br>17.99 (1.65)<br>-0.73 (0.57)	4.63 (0.52)<br>0.58 (0.09)<br>0.75 (0.03)<br>10.25 (1.40)<br>-0.45 (0.40)	4.50 (0.46)<br>11.83 (0.51)<br>0.75 (0.02)<br>-23.93 (2.72)<br>-0.20 (0.35)	21.36 (6.07)<br>12.17 (2.24)<br>1.26 (0.32)<br>-39.36 (13.09)<br>-13.89 (6.56)

medRxiv preprint doi: https://doi.org/10.1101/2025.04.23.25326206; this version posted April 25, 2025. The copyright holder for this preprint (which was not certified by peer review) is the author/funder, who has granted medRxiv a license to display the preprint in perpetuity.
This article is a US Government work. It is not subject to copyright under 17 USC 105 and is also made available for use under a CC0 license.

Supplement H: Ventral Diencephalon Predicted Brain Age

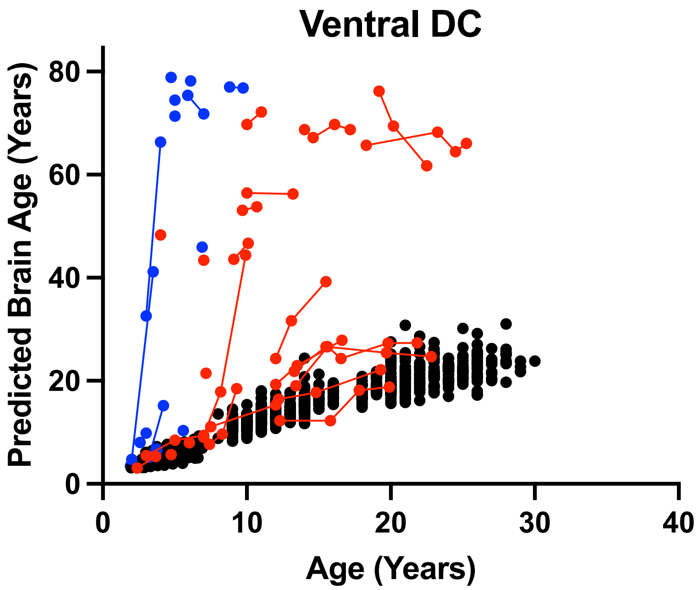

Figure H1. The relationship between predicted brain age and biological age in the Ventral Diencephalon (DC) plotted against chronological age. Late-infantile (n = 15, 20 total MRI scans) GM1 gangliosidosis patients are shown in blue, juvenile (n = 26, 61 total MRI scans) GM1 gangliosidosis patients are shown in red, and neurotypical controls (n = 556, 897 total MRI scans) participants are shown in black. Connecting lines indicate repeated scans on the same participant. Statistical analysis was performed using a linear mixed effects model to test the interactions between chronological age and cohort on predicted brain age and the results are summarized in Table I, with the estimated and standard errors shown in Table G1 of the Supplementary Materials.

medRxiv preprint doi: https://doi.org/10.1101/2025.04.23.25326206; this version posted April 25, 2025. The copyright holder for this preprint (which was not certified by peer review) is the author/funder, who has granted medRxiv a license to display the preprint in perpetuity. This article is a US Government work. It is not subject to copyright under 17 USC 105 and is also made available for use under a CC0 license.

Supplement I: Longitudinal Brain structures age gap estimation (BSAGE)

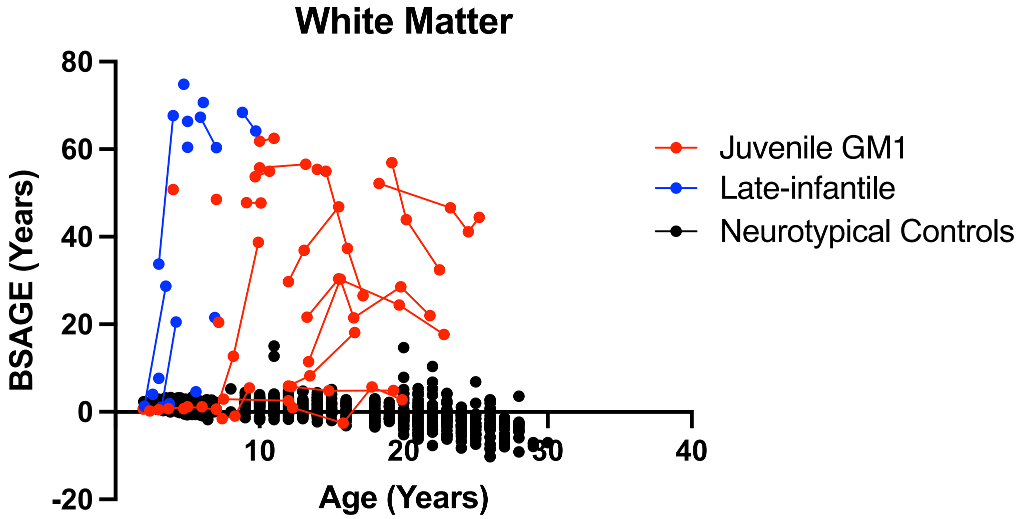

Figure I1. The longitudinal relationship between white matter BSAGE and chronological age. Late-infantile GM1 gangliosidosis patients are shown in blue, juvenile GM1 gangliosidosis patients are shown in red, and neurotypical controls participants are shown in black. Connecting lines indicate repeated scans on the same participant.

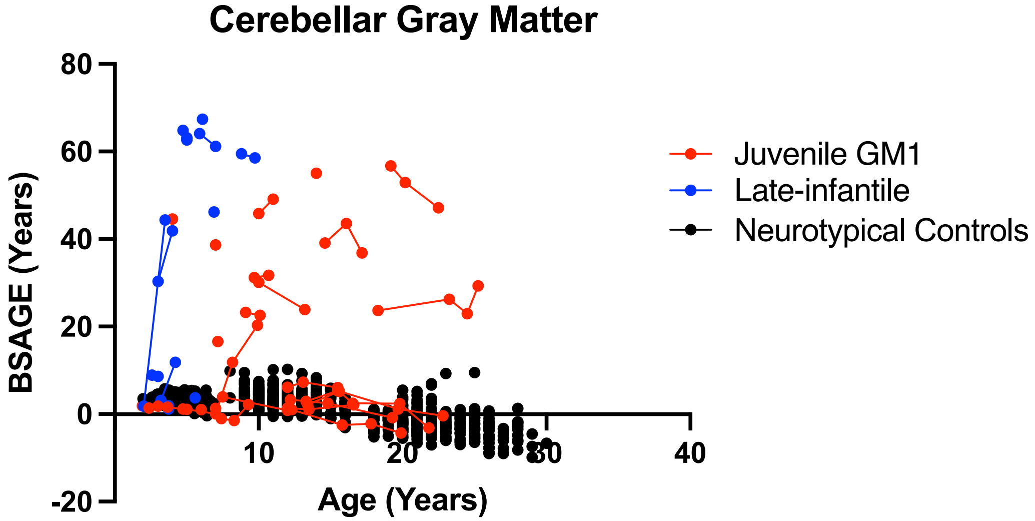

Figure I2. The longitudinal relationship between cerebellar gray matter BSAGE and chronological age. Late-infantile GM1 gangliosidosis patients are shown in blue, juvenile GM1 gangliosidosis patients are shown in red, and neurotypical controls participants are shown in black. Connecting lines indicate repeated scans on the same participant.

medRxiv preprint doi: https://doi.org/10.1101/2025.04.23.25326206; this version posted April 25, 2025. The copyright holder for this preprint
(which was not certified by peer review) is the author/funder, who has granted medRxiv a license to display the preprint in perpetuity.
This article is a US Government work. It is not subject to copyright under 17 USC 105 and is also made available
for use under a CC0 license.

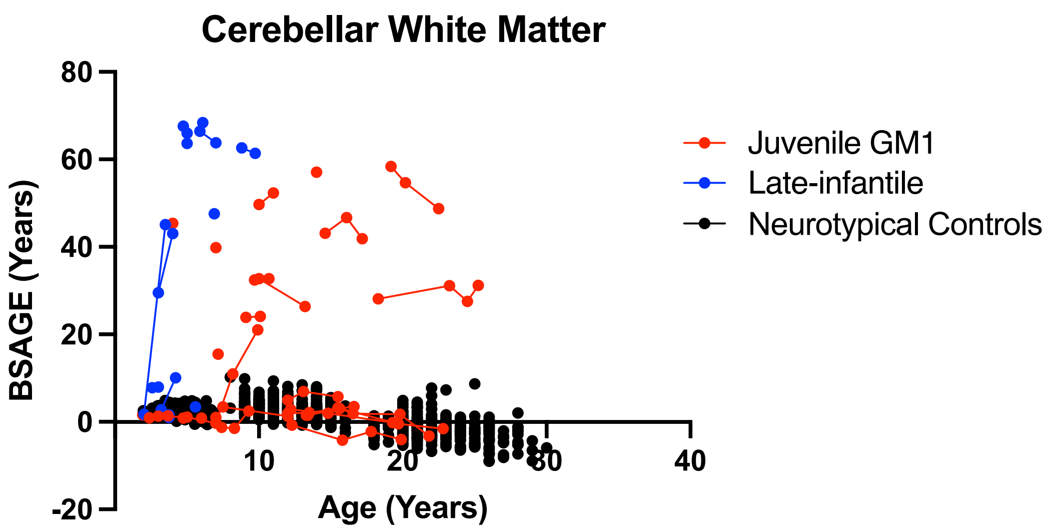

Figure I3. The longitudinal relationship between cerebellar white matter BSAGE and chronological age. Late-infantile GM1 gangliosidosis patients are shown in blue, juvenile GM1 gangliosidosis patients are shown in red, and neurotypical controls participants are shown in black. Connecting lines indicate repeated scans on the same participant.

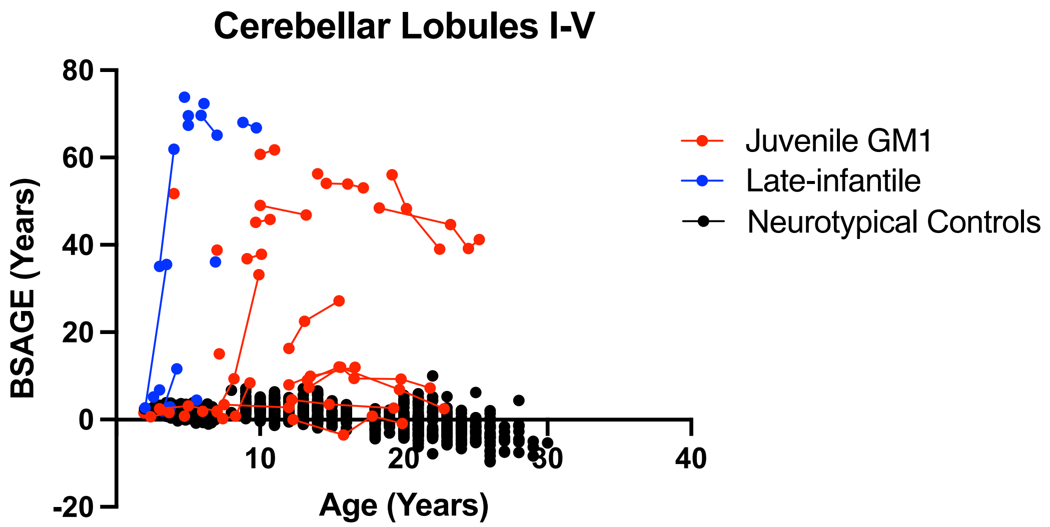

Figure I4. The longitudinal relationship between cerebellar lobules I-V BSAGE and chronological age. Late-infantile GM1 gangliosidosis patients are shown in blue, juvenile GM1 gangliosidosis patients are shown in red, and neurotypical controls participants are shown in black. Connecting lines indicate repeated scans on the same participant.

medRxiv preprint doi: https://doi.org/10.1101/2025.04.23.25326206; this version posted April 25, 2025. The copyright holder for this preprint
(which was not certified by peer review) is the author/funder, who has granted medRxiv a license to display the preprint in perpetuity.
This article is a US Government work. It is not subject to copyright under 17 USC 105 and is also made available
for use under a CC0 license.

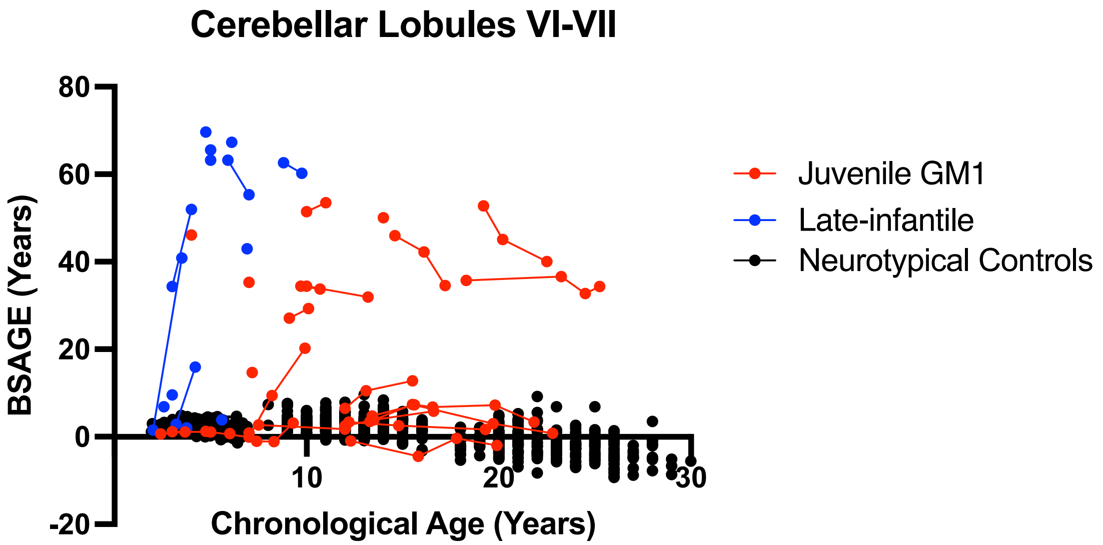

Figure I5. The longitudinal relationship between cerebellar lobules VI-VII BSAGE and chronological age. Late-infantile GM1 gangliosidosis patients are shown in blue, juvenile GM1 gangliosidosis patients are shown in red, and neurotypical controls participants are shown in black. Connecting lines indicate repeated scans on the same participant.

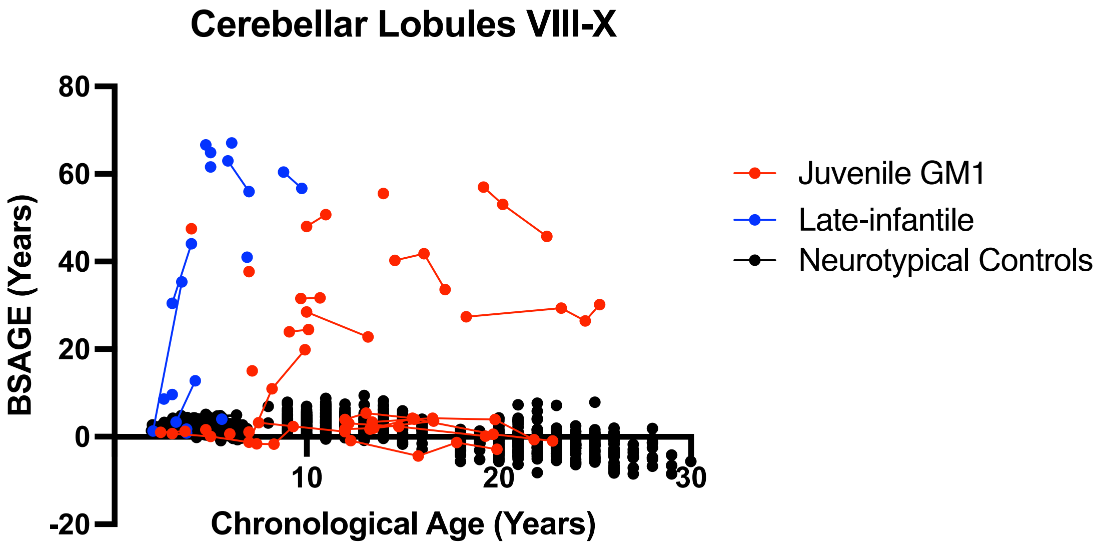

Figure I6. The longitudinal relationship between cerebellar lobules VIII-X BSAGE and chronological age. Late-infantile GM1 gangliosidosis patients are shown in blue, juvenile GM1 gangliosidosis patients are shown in red, and neurotypical controls participants are shown in black. Connecting lines indicate repeated scans on the same participant.

medRxiv preprint doi: https://doi.org/10.1101/2025.04.23.25326206; this version posted April 25, 2025. The copyright holder for this preprint (which was not certified by peer review) is the author/funder, who has granted medRxiv a license to display the preprint in perpetuity. This article is a US Government work. It is not subject to copyright under 17 USC 105 and is also made available for use under a CC0 license.

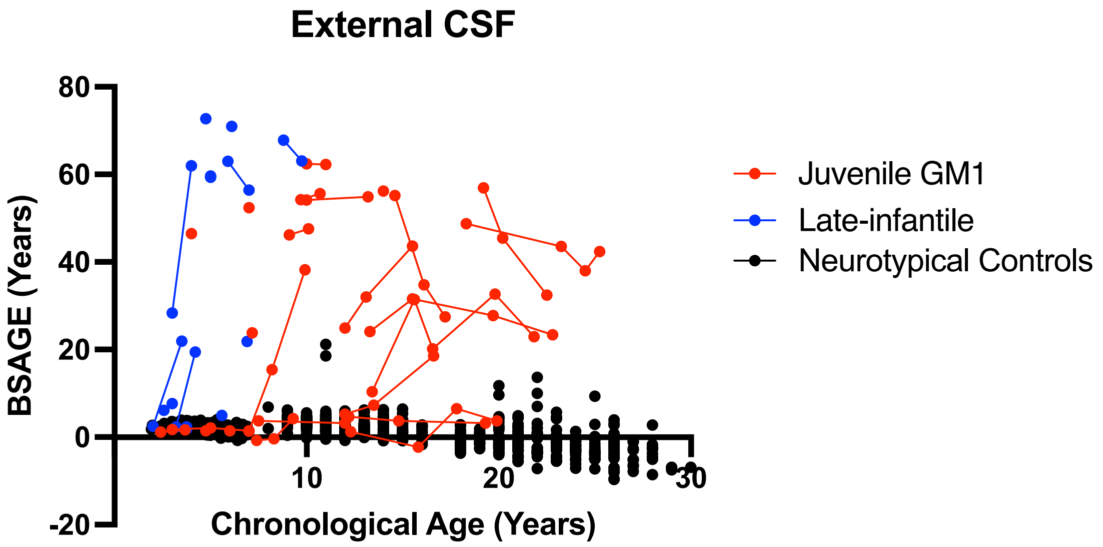

Figure I7. The longitudinal relationship between external cerebrospinal fluid (CSF) BSAGE and chronological age. Late-infantile GM1 gangliosidosis patients are shown in blue, juvenile GM1 gangliosidosis patients are shown in red, and neurotypical controls participants are shown in black. Connecting lines indicate repeated scans on the same participant.

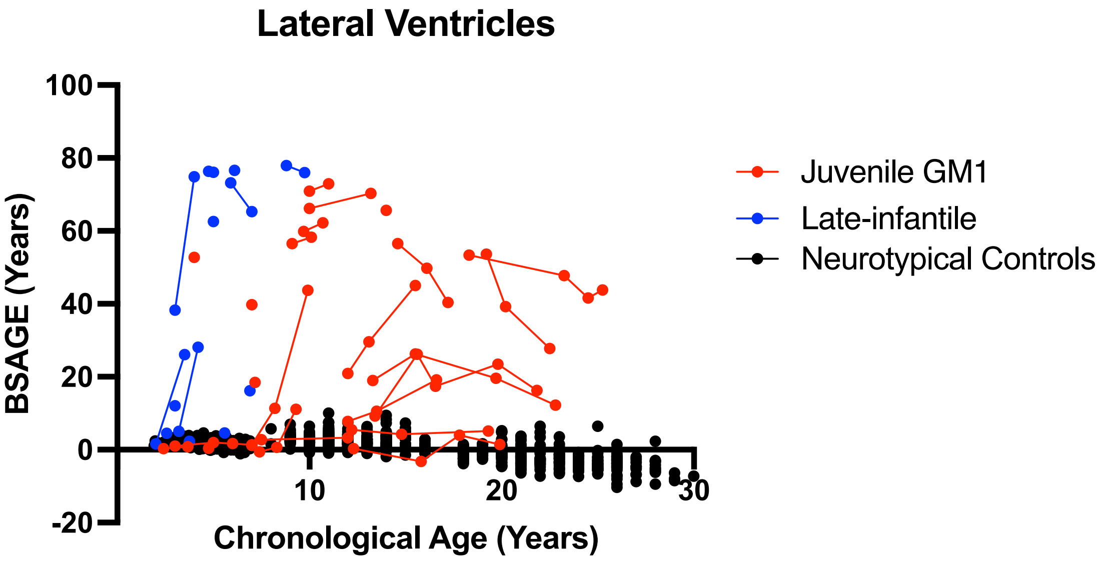

Figure I8. The longitudinal relationship between lateral ventricles BSAGE and chronological age. Late-infantile GM1 gangliosidosis patients are shown in blue, juvenile GM1 gangliosidosis patients are shown in red, and neurotypical controls participants are shown in black. Connecting lines indicate repeated scans on the same participant.

medRxiv preprint doi: https://doi.org/10.1101/2025.04.23.25326206; this version posted April 25, 2025. The copyright holder for this preprint (which was not certified by peer review) is the author/funder, who has granted medRxiv a license to display the preprint in perpetuity.
This article is a US Government work. It is not subject to copyright under 17 USC 105 and is also made available for use under a CC0 license.

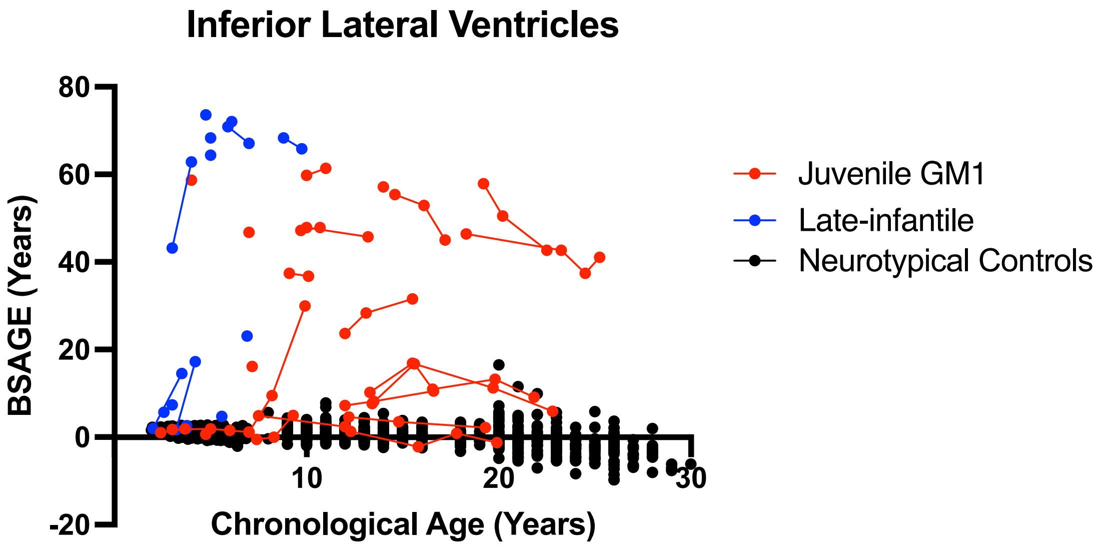

Figure I9. The longitudinal relationship between inferior lateral ventricles BSAGE and chronological age. Late-infantile GM1 gangliosidosis patients are shown in blue, juvenile GM1 gangliosidosis patients are shown in red, and neurotypical controls participants are shown in black. Connecting lines indicate repeated scans on the same participant.

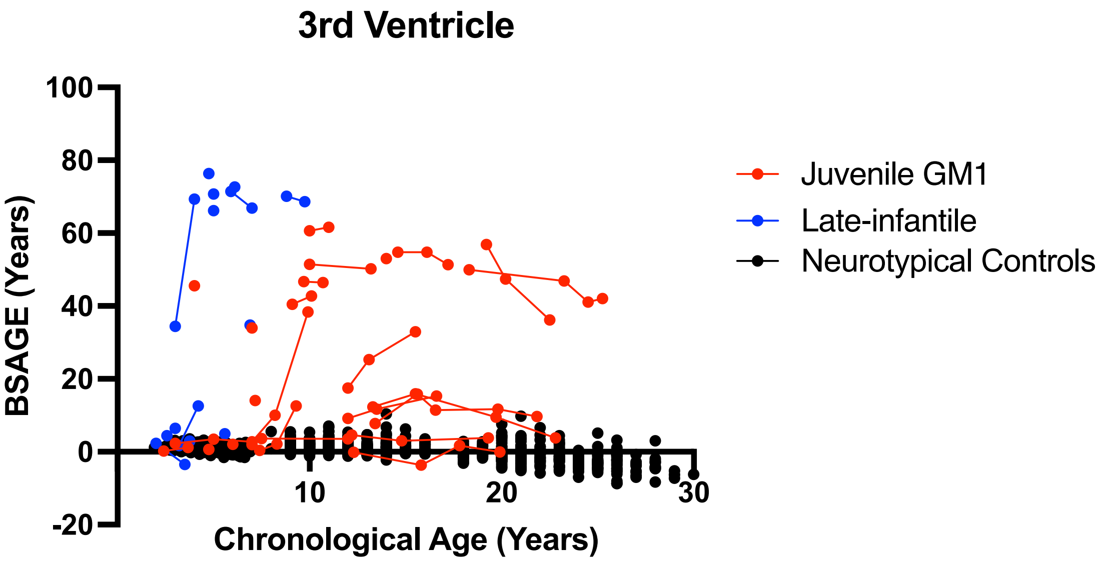

Figure I10. The longitudinal relationship between 3rd ventricle BSAGE and chronological age. Late-infantile GM1 gangliosidosis patients are shown in blue, juvenile GM1 gangliosidosis patients are shown in red, and neurotypical controls participants are shown in black. Connecting lines indicate repeated scans on the same participant.

medRxiv preprint doi: https://doi.org/10.1101/2025.04.23.25326206; this version posted April 25, 2025. The copyright holder for this preprint (which was not certified by peer review) is the author/funder, who has granted medRxiv a license to display the preprint in perpetuity.
This article is a US Government work. It is not subject to copyright under 17 USC 105 and is also made available for use under a CC0 license.

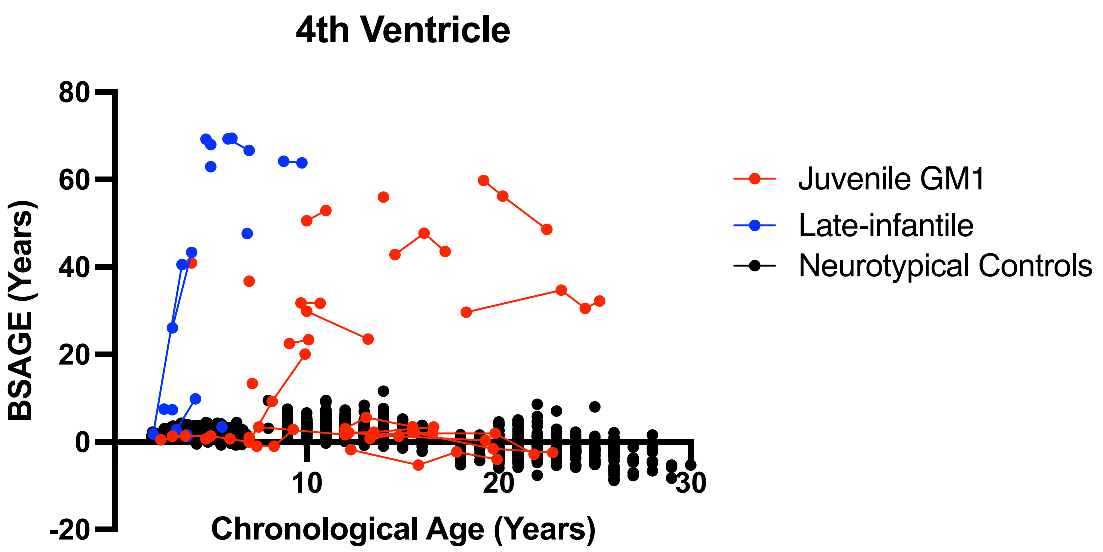

Figure I11. The longitudinal relationship between 4th ventricle BSAGE and chronological age. Late-infantile GM1 gangliosidosis patients are shown in blue, juvenile GM1 gangliosidosis patients are shown in red, and neurotypical controls participants are shown in black. Connecting lines indicate repeated scans on the same participant.

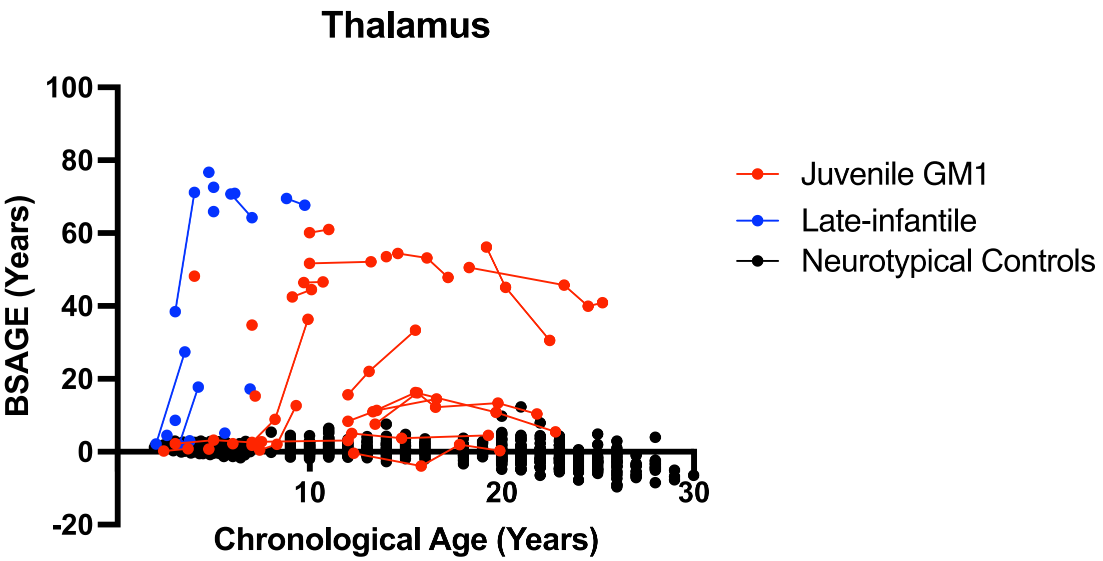

Figure I12. The longitudinal relationship between thalamus BSAGE and chronological age. Late-infantile GM1 gangliosidosis patients are shown in blue, juvenile GM1 gangliosidosis patients are shown in red, and neurotypical controls participants are shown in black. Connecting lines indicate repeated scans on the same participant.

medRxiv preprint doi: https://doi.org/10.1101/2025.04.23.25326206; this version posted April 25, 2025. The copyright holder for this preprint (which was not certified by peer review) is the author/funder, who has granted medRxiv a license to display the preprint in perpetuity.
This article is a US Government work. It is not subject to copyright under 17 USC 105 and is also made available for use under a CC0 license.

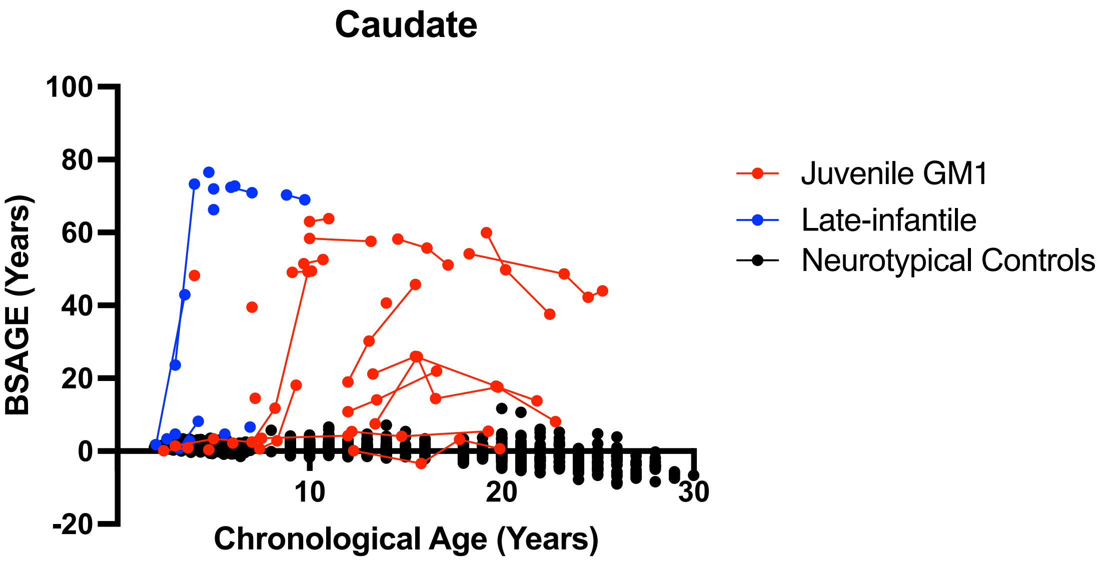

Figure I13. The longitudinal relationship between caudate nucleus BSAGE and chronological age. Late-infantile GM1 gangliosidosis patients are shown in blue, juvenile GM1 gangliosidosis patients are shown in red, and neurotypical controls participants are shown in black. Connecting lines indicate repeated scans on the same participant.

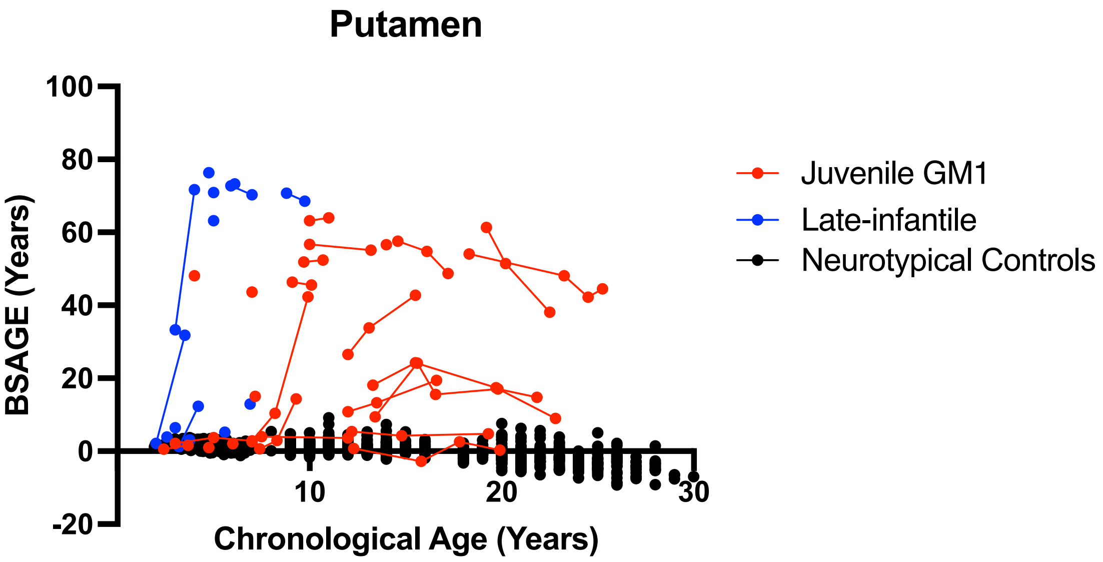

Figure I14. The longitudinal relationship between putamen BSAGE and chronological age. Late-infantile GM1 gangliosidosis patients are shown in blue, juvenile GM1 gangliosidosis patients are shown in red, and neurotypical controls participants are shown in black. Connecting lines indicate repeated scans on the same participant.

medRxiv preprint doi: https://doi.org/10.1101/2025.04.23.25326206; this version posted April 25, 2025. The copyright holder for this preprint (which was not certified by peer review) is the author/funder, who has granted medRxiv a license to display the preprint in perpetuity. This article is a US Government work. It is not subject to copyright under 17 USC 105 and is also made available for use under a CC0 license.

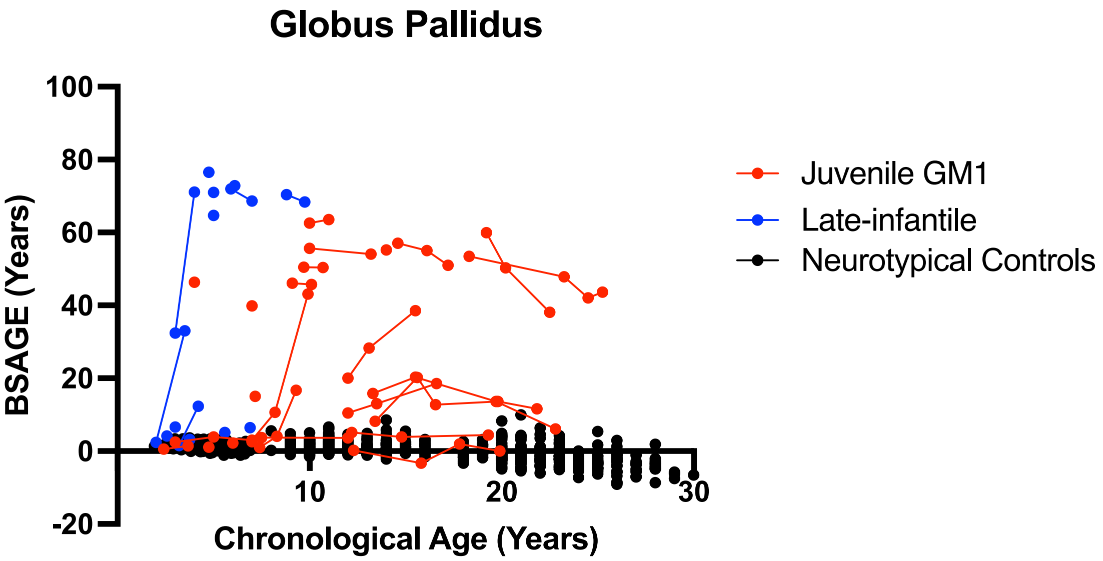

Figure I15. The longitudinal relationship between globus pallidus BSAGE and chronological age. Late-infantile GM1 gangliosidosis patients are shown in blue, juvenile GM1 gangliosidosis patients are shown in red, and neurotypical controls participants are shown in black. Connecting lines indicate repeated scans on the same participant.

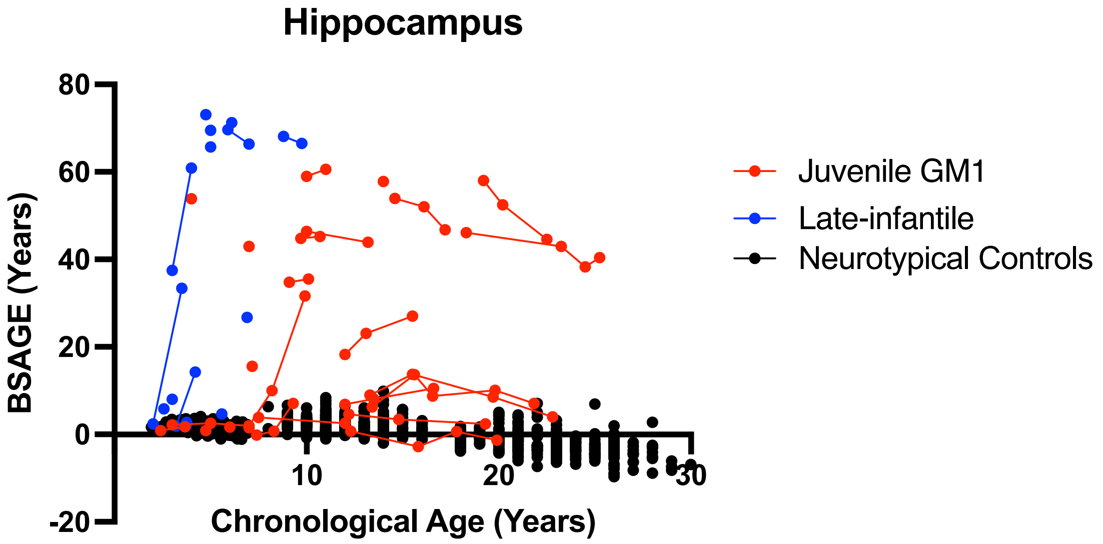

Figure I16. The longitudinal relationship between hippocampus BSAGE and chronological age. Late-infantile GM1 gangliosidosis patients are shown in blue, juvenile GM1 gangliosidosis patients are shown in red, and neurotypical controls participants are shown in black. Connecting lines indicate repeated scans on the same participant.

medRxiv preprint doi: https://doi.org/10.1101/2025.04.23.25326206; this version posted April 25, 2025. The copyright holder for this preprint (which was not certified by peer review) is the author/funder, who has granted medRxiv a license to display the preprint in perpetuity.
This article is a US Government work. It is not subject to copyright under 17 USC 105 and is also made available for use under a CC0 license.

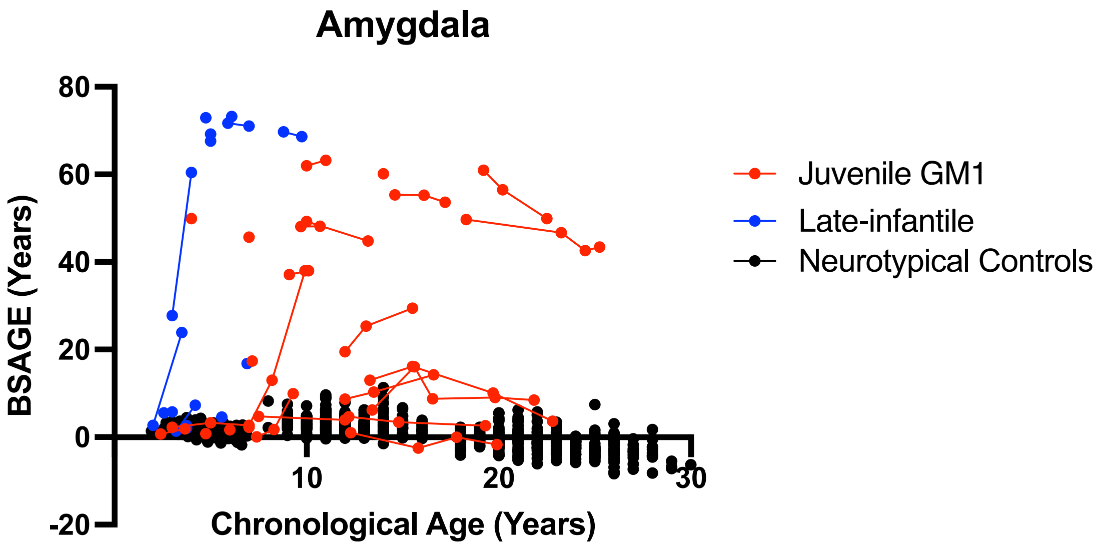

Figure I17. The longitudinal relationship between amygdala BSAGE and chronological age. Late-infantile GM1 gangliosidosis patients are shown in blue, juvenile GM1 gangliosidosis patients are shown in red, and neurotypical controls participants are shown in black. Connecting lines indicate repeated scans on the same participant.

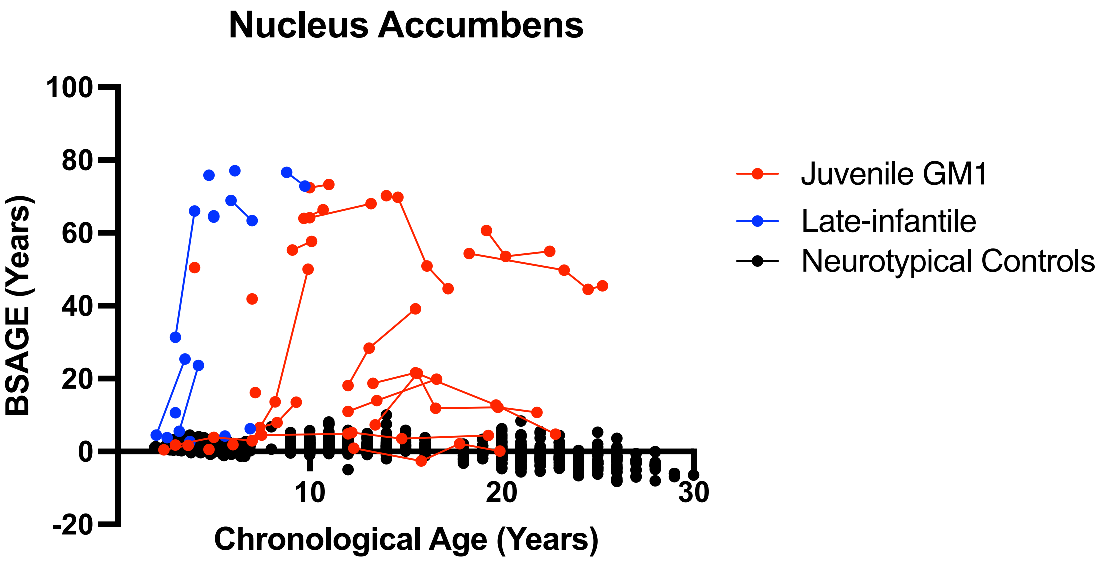

Figure I18. The longitudinal relationship between nucleus accumbens BSAGE and chronological age. Late-infantile GM1 gangliosidosis patients are shown in blue, juvenile GM1 gangliosidosis patients are shown in red, and neurotypical controls participants are shown in black. Connecting lines indicate repeated scans on the same participant.

medRxiv preprint doi: https://doi.org/10.1101/2025.04.23.25326206; this version posted April 25, 2025. The copyright holder for this preprint
(which was not certified by peer review) is the author/funder, who has granted medRxiv a license to display the preprint in perpetuity.
This article is a US Government work. It is not subject to copyright under 17 USC 105 and is also made available
for use under a CC0 license.

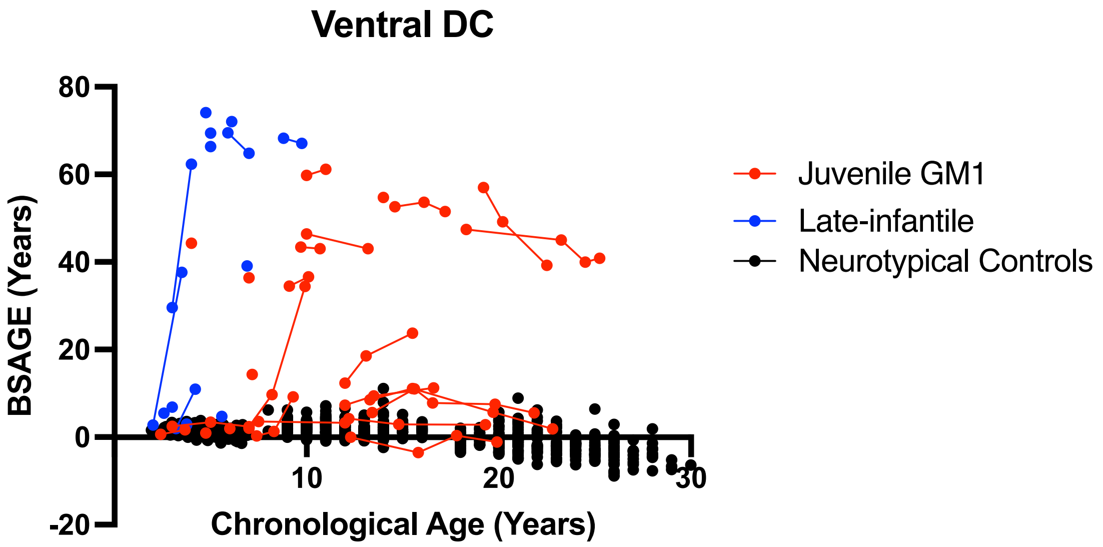

Figure I19. The longitudinal relationship between ventral diencephalon (DC) BSAGE and chronological age. Late-infantile GM1 gangliosidosis patients are shown in blue, juvenile GM1 gangliosidosis patients are shown in red, and neurotypical controls participants are shown in black. Connecting lines indicate repeated scans on the same participant.

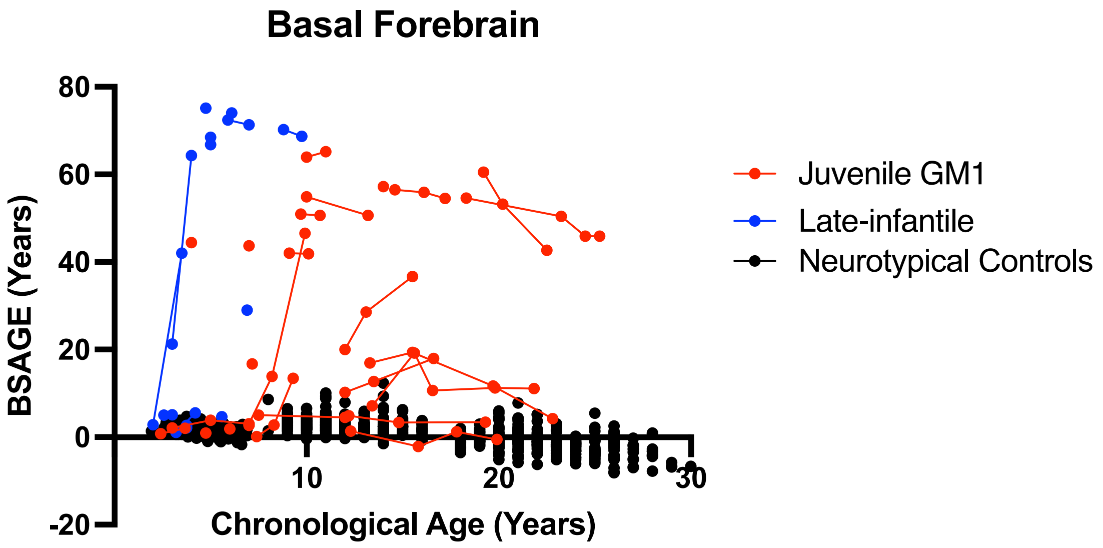

Figure I20. The longitudinal relationship between basal forebrain BSAGE and chronological age. Late-infantile GM1 gangliosidosis patients are shown in blue, juvenile GM1 gangliosidosis patients are shown in red, and neurotypical controls participants are shown in black. Connecting lines indicate repeated scans on the same participant.

medRxiv preprint doi: https://doi.org/10.1101/2025.04.23.25326206; this version posted April 25, 2025. The copyright holder for this preprint (which was not certified by peer review) is the author/funder, who has granted medRxiv a license to display the preprint in perpetuity. This article is a US Government work. It is not subject to copyright under 17 USC 105 and is also made available for use under a CC0 license.

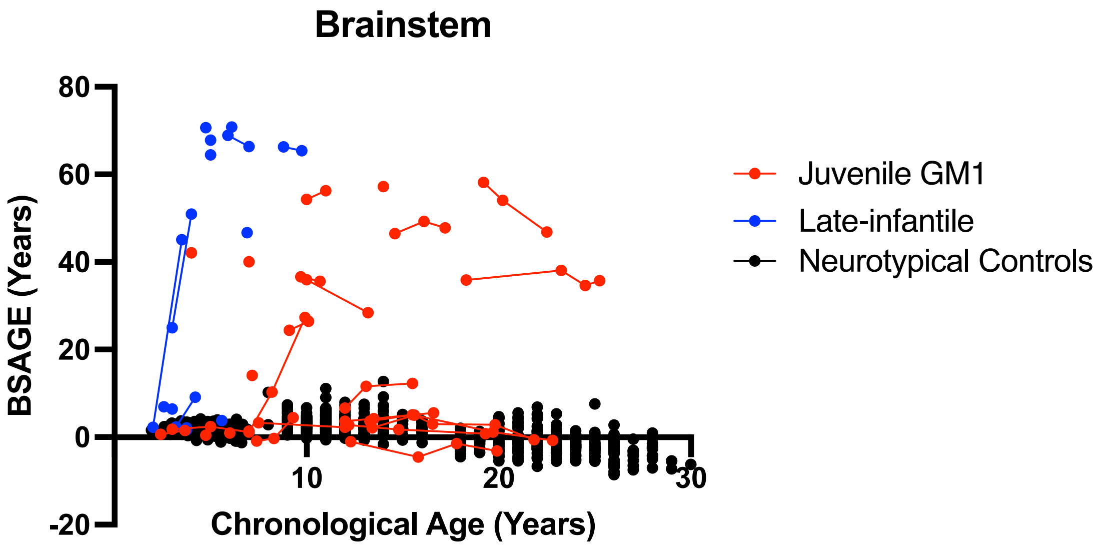

Figure I21. The longitudinal relationship between brainstem BSAGE and chronological age. Late-infantile GM1 gangliosidosis patients are shown in blue, juvenile GM1 gangliosidosis patients are shown in red, and neurotypical controls participants are shown in black. Connecting lines indicate repeated scans on the same participant.

medRxiv preprint doi: https://doi.org/10.1101/2025.04.23.25326206; this version posted April 25, 2025. The copyright holder for this preprint (which was not certified by peer review) is the author/funder, who has granted medRxiv a license to display the preprint in perpetuity. This article is a US Government work. It is not subject to copyright under 17 USC 105 and is also made available for use under a CC0 license.

Supplement J: Cross-Sectional Brain structures age gap estimation (BSAGE)

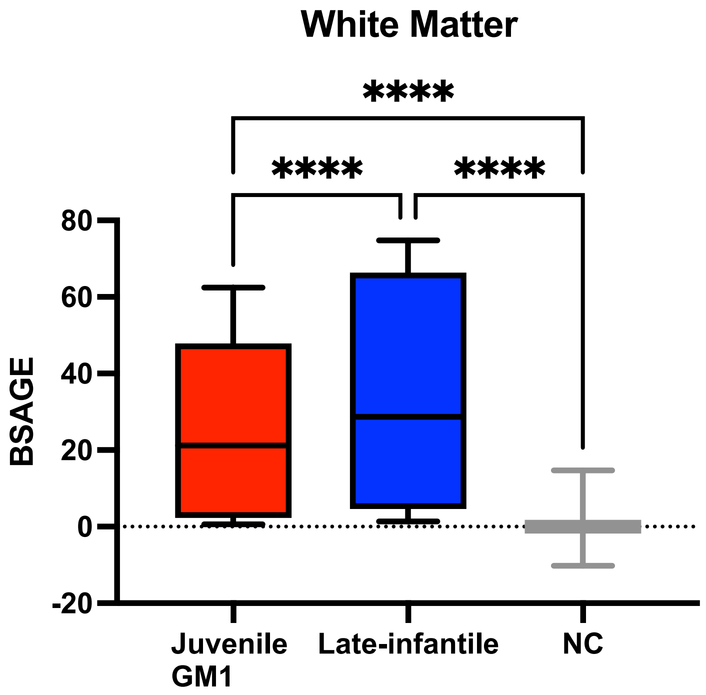

Figure J1. Comparison of White Matter BSAGE between Juvenile GM1 patients, late-infantile GM1 patients, and neurotypical controls. Late-infantile GM1 patients had an average BSAGE = 36.99, juvenile GM1 patients had an average BSAGE = 25.57, and NC participants had an average BSAGE = -0.21. An analysis of variance (ANOVA) showed statistically significant ($F(2, 595) = 358.8$, $p < 0.0001$) differences in BSAGE between the cohorts. A post-hoc Tukey test showed the late-infantile cohort had a statistically significantly higher BSAGE compared to both the neurotypical controls ($p < 0.0001$) and juvenile GM1 patients ($p < 0.0001$). The juvenile GM1 cohort also had a significantly higher BSAGE compared to the neurotypical controls ($p < 0.0001$).

medRxiv preprint doi: https://doi.org/10.1101/2025.04.23.25326206; this version posted April 25, 2025. The copyright holder for this preprint (which was not certified by peer review) is the author/funder, who has granted medRxiv a license to display the preprint in perpetuity. This article is a US Government work. It is not subject to copyright under 17 USC 105 and is also made available for use under a CC0 license.

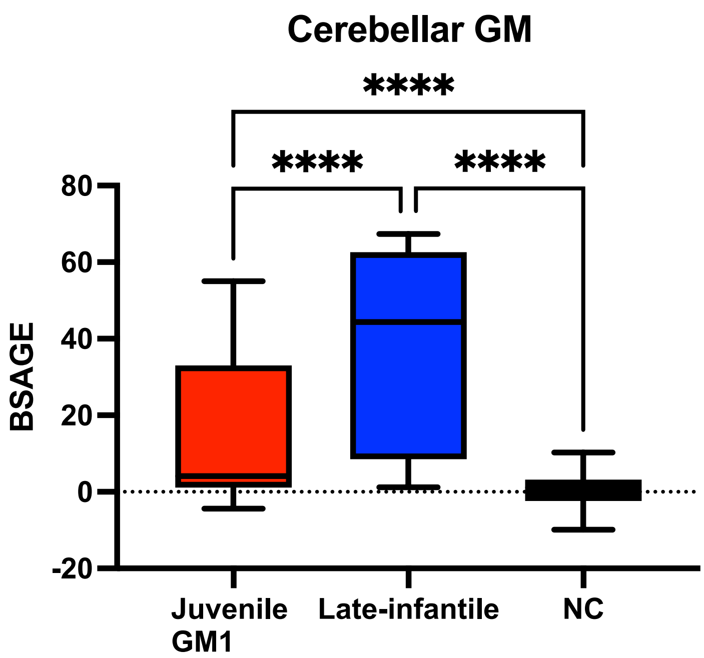

Figure J2. Comparison of Cerebellar Gray Matter BSAGE between Juvenile GM1 patients, late-infantile GM1 patients, and neurotypical controls. Late-infantile GM1 patients had an average BSAGE = 36.41, juvenile GM1 patients had an average BSAGE = 16.42, and NC participants had an average BSAGE = 0.49. An analysis of variance (ANOVA) showed statistically significant ($F(2, 595) = 270.8$, $p < 0.0001$) differences in BSAGE between the cohorts. A post-hoc Tukey test showed the late-infantile cohort had a statistically significantly higher BSAGE compared to both the neurotypical controls ($p < 0.0001$) and juvenile GM1 patients ($p < 0.0001$). The juvenile GM1 cohort also had a significantly higher BSAGE compared to the neurotypical controls ($p < 0.0001$).

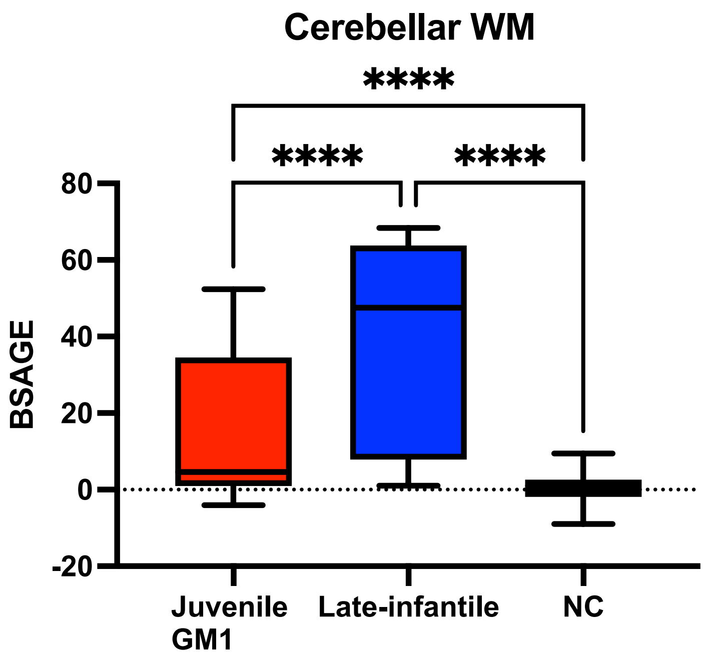

medRxiv preprint doi: https://doi.org/10.1101/2025.04.23.25326206; this version posted April 25, 2025. The copyright holder for this preprint (which was not certified by peer review) is the author/funder, who has granted medRxiv a license to display the preprint in perpetuity. This article is a US Government work. It is not subject to copyright under 17 USC 105 and is also made available for use under a CC0 license.

Figure J3. Comparison of Cerebellar White Matter BSAGE between Juvenile GM1 patients, late-infantile GM1 patients, and neurotypical controls. Late-infantile GM1 patients had an average BSAGE = 38.03, juvenile GM1 patients had an average BSAGE = 16.75, and NC participants had an average BSAGE = 0.36. An analysis of variance (ANOVA) showed statistically significant ($F(2, 595) = 285.9$, $p < 0.0001$) differences in BSAGE between the cohorts. A post-hoc Tukey test showed the late-infantile cohort had a statistically significantly higher BSAGE compared to both the neurotypical controls ($p < 0.0001$) and juvenile GM1 patients ($p < 0.0001$). The juvenile GM1 cohort also had a significantly higher BSAGE compared to the neurotypical controls ($p < 0.0001$).

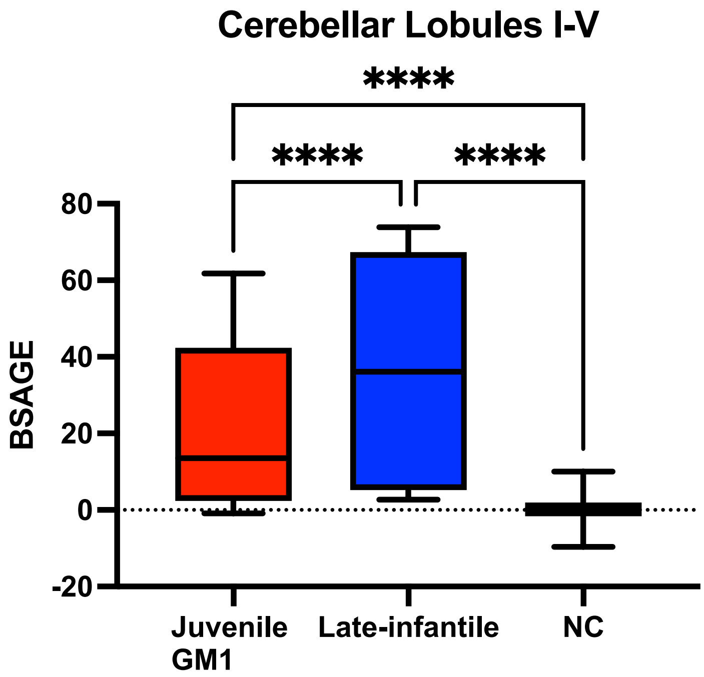

Figure J4. Comparison of Cerebellar Lobules I-V BSAGE between Juvenile GM1 patients, late-infantile GM1 patients, and neurotypical controls. Late-infantile GM1 patients had an average BSAGE = 38.82, juvenile GM1 patients had an average BSAGE = 22.79, and NC participants had an average BSAGE = 0.16. An analysis of variance (ANOVA) showed statistically significant ($F(2, 595) = 585.6$, $p < 0.0001$) differences in BSAGE between the cohorts. A post-hoc Tukey test showed the late-infantile cohort had a statistically significantly higher BSAGE compared to both the neurotypical controls ($p < 0.0001$) and juvenile GM1 patients ($p < 0.0001$). The juvenile GM1 cohort also had a significantly higher BSAGE compared to the neurotypical controls ($p < 0.0001$).

medRxiv preprint doi: https://doi.org/10.1101/2025.04.23.25326206; this version posted April 25, 2025. The copyright holder for this preprint
(which was not certified by peer review) is the author/funder, who has granted medRxiv a license to display the preprint in perpetuity.
This article is a US Government work. It is not subject to copyright under 17 USC 105 and is also made available
for use under a CC0 license.

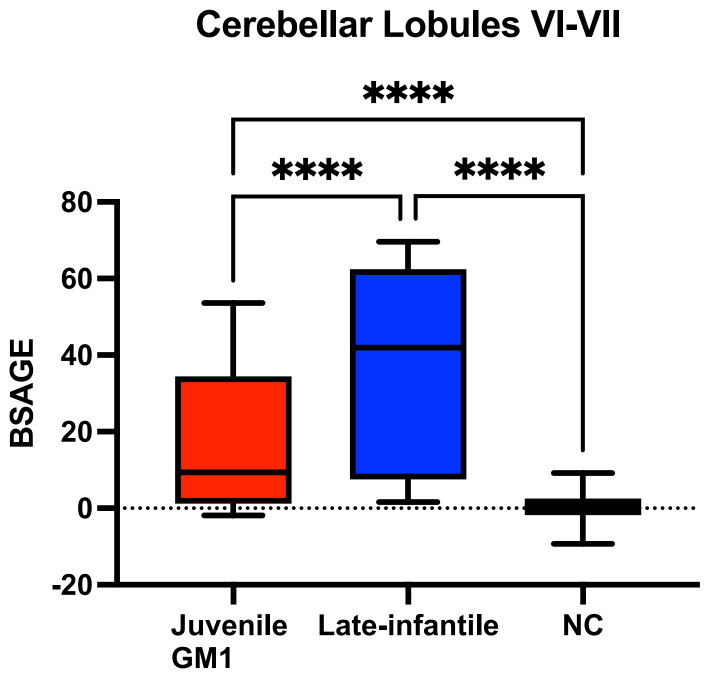

Figure J5. Comparison of Cerebellar Lobules VI-VII BSAGE between Juvenile GM1 patients, late-infantile GM1 patients, and neurotypical controls. Late-infantile GM1 patients had an average BSAGE = 36.97, juvenile GM1 patients had an average BSAGE = 17.58, and NC participants had an average BSAGE = 0.35. An analysis of variance (ANOVA) showed statistically significant ($F(2, 595) = 358.3$, $p < 0.0001$) differences in BSAGE between the cohorts. A post-hoc Tukey test showed the late-infantile cohort had a statistically significantly higher BSAGE compared to both the neurotypical controls ($p < 0.0001$) and juvenile GM1 patients ($p < 0.0001$). The juvenile GM1 cohort also had a significantly higher BSAGE compared to the neurotypical controls ($p < 0.0001$).

medRxiv preprint doi: https://doi.org/10.1101/2025.04.23.25326206; this version posted April 25, 2025. The copyright holder for this preprint
(which was not certified by peer review) is the author/funder, who has granted medRxiv a license to display the preprint in perpetuity.
This article is a US Government work. It is not subject to copyright under 17 USC 105 and is also made available
for use under a CC0 license.

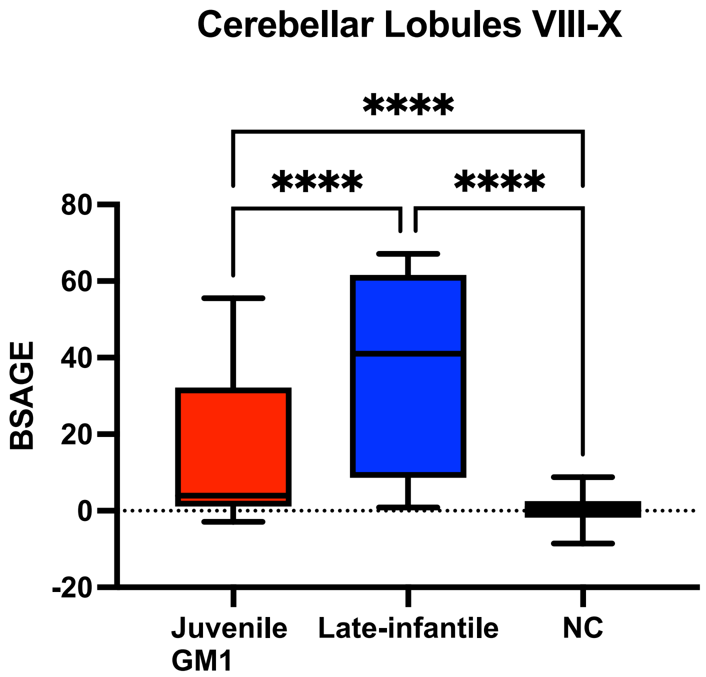

Figure J6. Comparison of Cerebellar Lobules VIII-X BSAGE between Juvenile GM1 patients, late-infantile GM1 patients, and neurotypical controls. Late-infantile GM1 patients had an average BSAGE = 35.40, juvenile GM1 patients had an average BSAGE = 16.50, and NC participants had an average BSAGE = 0.40. An analysis of variance (ANOVA) showed statistically significant ($F(2, 595) = 284.5$, $p < 0.0001$) differences in BSAGE between the cohorts. A post-hoc Tukey test showed the late-infantile cohort had a statistically significantly higher BSAGE compared to both the neurotypical controls ($p < 0.0001$) and juvenile GM1 patients ($p < 0.0001$). The juvenile GM1 cohort also had a significantly higher BSAGE compared to the neurotypical controls ($p < 0.0001$).

medRxiv preprint doi: https://doi.org/10.1101/2025.04.23.25326206; this version posted April 25, 2025. The copyright holder for this preprint (which was not certified by peer review) is the author/funder, who has granted medRxiv a license to display the preprint in perpetuity.
This article is a US Government work. It is not subject to copyright under 17 USC 105 and is also made available for use under a CC0 license.

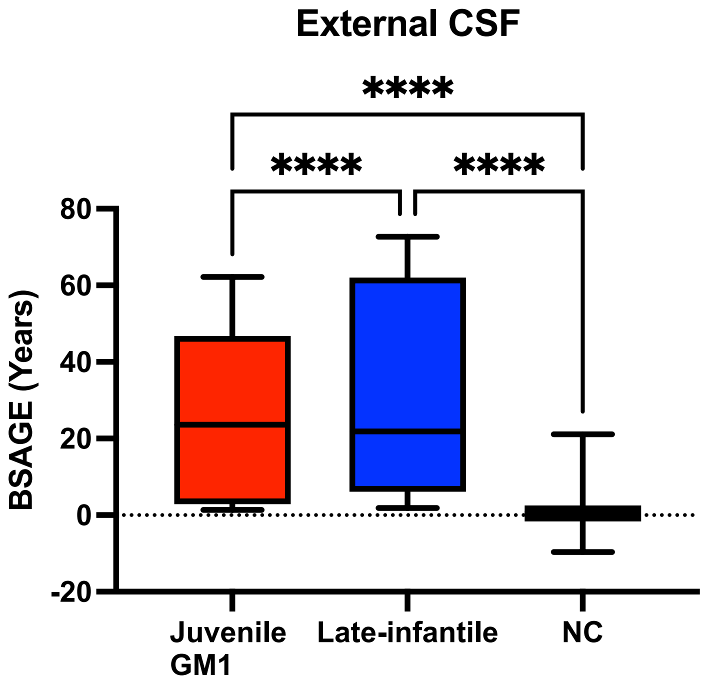

Figure J7. Comparison of External Cerebrospinal Fluid (CSF) BSAGE between Juvenile GM1 patients, late-infantile GM1 patients, and neurotypical controls (NC). Late-infantile GM1 patients had an average BSAGE = 35.35, juvenile GM1 patients had an average BSAGE = 25.87, and NC participants had an average BSAGE = 0.46. An analysis of variance (ANOVA) showed statistically significant ($F(2, 595) = 428.0$, $p < 0.0001$) differences in BSAGE between the cohorts. A post-hoc Tukey test showed the late-infantile cohort had a statistically significantly higher BSAGE compared to both the neurotypical controls ($p < 0.0001$) and juvenile GM1 patients ($p < 0.0001$). The juvenile GM1 cohort also had a significantly higher BSAGE compared to the neurotypical controls ($p < 0.0001$).

medRxiv preprint doi: https://doi.org/10.1101/2025.04.23.25326206; this version posted April 25, 2025. The copyright holder for this preprint
(which was not certified by peer review) is the author/funder, who has granted medRxiv a license to display the preprint in perpetuity.
This article is a US Government work. It is not subject to copyright under 17 USC 105 and is also made available
for use under a CC0 license.

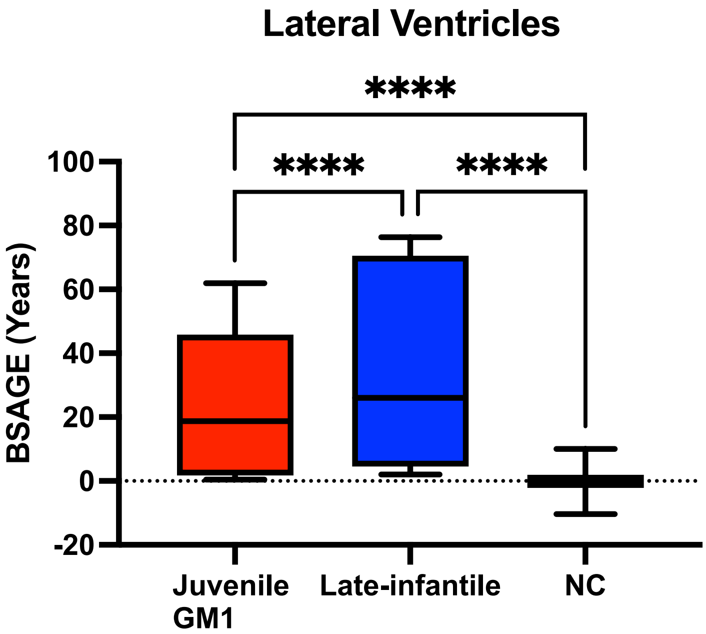

Figure J8. Comparison of lateral ventricle BSAGE between Juvenile GM1 patients, late-infantile GM1 patients, and neurotypical controls (NC). Late-infantile GM1 patients had an average BSAGE = 37.63, juvenile GM1 patients had an average BSAGE = 25.14, and NC participants had an average BSAGE = -0.06. An analysis of variance (ANOVA) showed statistically significant ($F(2, 595) = 572.6$, $p < 0.0001$) differences in BSAGE between the cohorts. A post-hoc Tukey test showed the late-infantile cohort had a statistically significantly higher BSAGE compared to both the neurotypical controls ($p < 0.0001$) and juvenile GM1 patients ($p < 0.0001$). The juvenile GM1 cohort also had a significantly higher BSAGE compared to the neurotypical controls ($p < 0.0001$).

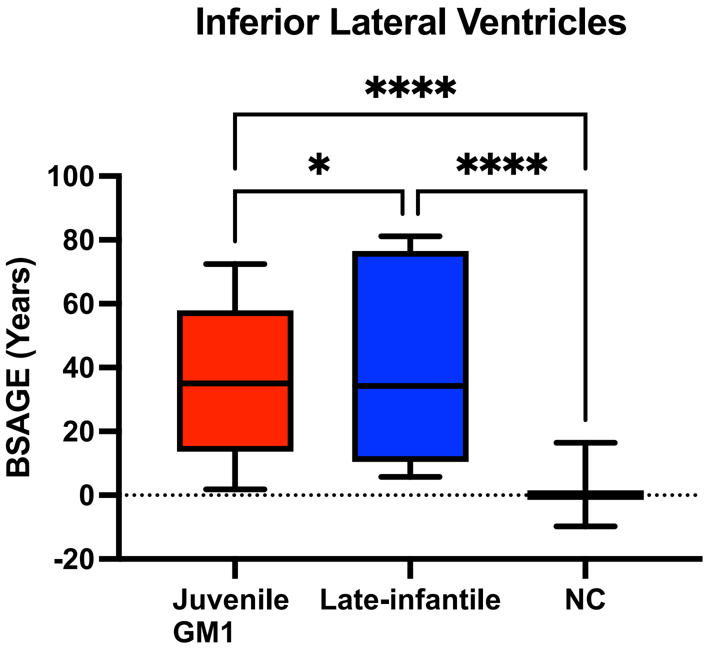

medRxiv preprint doi: https://doi.org/10.1101/2025.04.23.25326206; this version posted April 25, 2025. The copyright holder for this preprint (which was not certified by peer review) is the author/funder, who has granted medRxiv a license to display the preprint in perpetuity. This article is a US Government work. It is not subject to copyright under 17 USC 105 and is also made available for use under a CC0 license.

Figure J9. Comparison of the inferior lateral ventricle BSAGE between Juvenile GM1 patients, late-infantile GM1 patients, and neurotypical controls (NC). Late-infantile GM1 patients had an average BSAGE = 42.49, juvenile GM1 patients had an average BSAGE = 35.77, and NC participants had an average BSAGE = -0.09. An analysis of variance (ANOVA) showed statistically significant ($F(2, 595) = 673.1$, $p < 0.0001$) differences in BSAGE between the cohorts. A post-hoc Tukey test showed the late-infantile cohort had a statistically significantly higher BSAGE compared to both the neurotypical controls ($p < 0.0001$) and juvenile GM1 patients ($p < 0.0001$). The juvenile GM1 cohort also had a significantly higher BSAGE compared to the neurotypical controls ($p < 0.0001$).

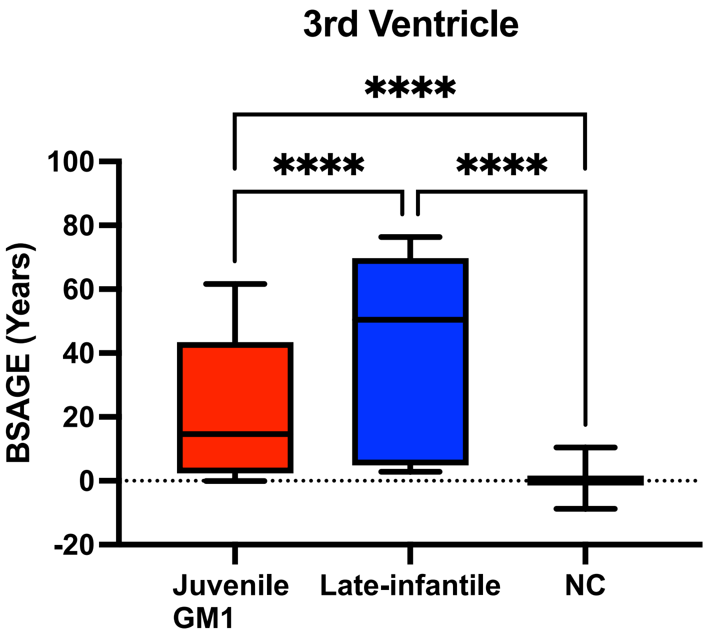

Figure J10. Comparison of the 3rd ventricle BSAGE between Juvenile GM1 patients, late-infantile GM1 patients, and neurotypical controls (NC). Late-infantile GM1 patients had an average BSAGE = 40.00, juvenile GM1 patients had an average BSAGE = 23.34, and NC participants had an average BSAGE = 0.04. An analysis of variance (ANOVA) showed statistically significant ($F(2,594) = 650.1$, $p < 0.0001$) differences in BSAGE between the cohorts. A post-hoc Tukey test showed the late-infantile cohort had a statistically significantly higher BSAGE compared to both the neurotypical controls ($p < 0.0001$) and juvenile GM1 patients ($p < 0.0001$). The juvenile GM1 cohort also had a significantly higher BSAGE compared to the neurotypical controls ($p < 0.0001$).

medRxiv preprint doi: https://doi.org/10.1101/2025.04.23.25326206; this version posted April 25, 2025. The copyright holder for this preprint (which was not certified by peer review) is the author/funder, who has granted medRxiv a license to display the preprint in perpetuity.
This article is a US Government work. It is not subject to copyright under 17 USC 105 and is also made available for use under a CC0 license.

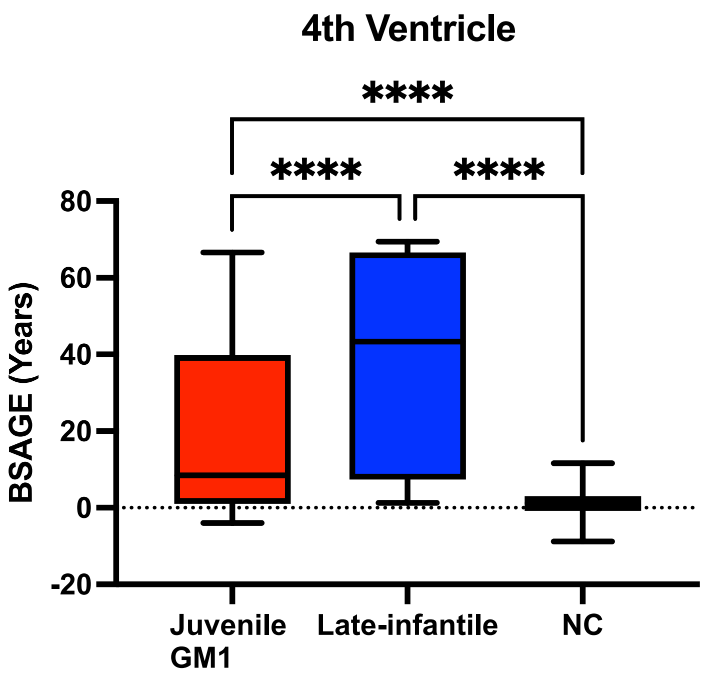

Figure J11. Comparison of the 4rd ventricle BSAGE between Juvenile GM1 patients, late-infantile GM1 patients, and neurotypical controls (NC). Late-infantile GM1 patients had an average BSAGE = 37.52, juvenile GM1 patients had an average BSAGE = 20.09, and NC participants had an average BSAGE = 0.96. An analysis of variance (ANOVA) showed statistically significant ($F(2, 595) = 438.0$, $p < 0.0001$) differences in BSAGE between the cohorts. A post-hoc Tukey test showed the late-infantile cohort had a statistically significantly higher BSAGE compared to both the neurotypical controls ($p < 0.0001$) and juvenile GM1 patients ($p < 0.0001$). The juvenile GM1 cohort also had a significantly higher BSAGE compared to the neurotypical controls ($p < 0.0001$).

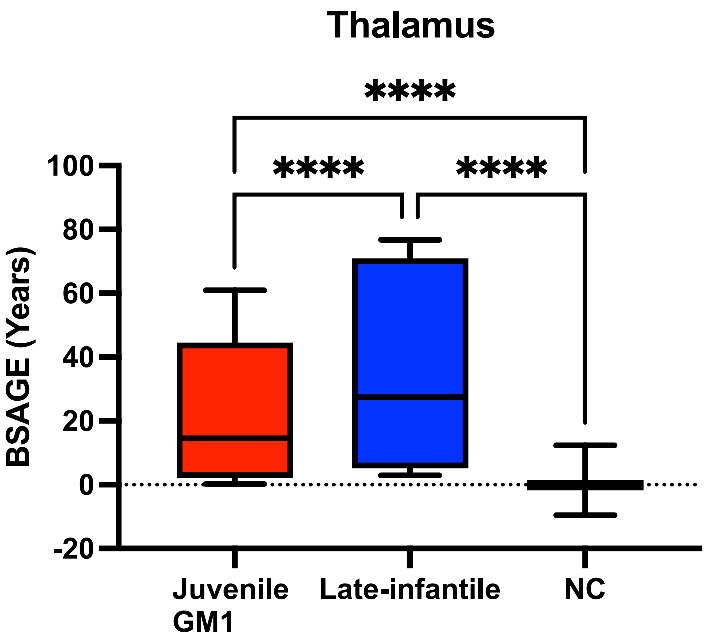

medRxiv preprint doi: https://doi.org/10.1101/2025.04.23.25326206; this version posted April 25, 2025. The copyright holder for this preprint (which was not certified by peer review) is the author/funder, who has granted medRxiv a license to display the preprint in perpetuity. This article is a US Government work. It is not subject to copyright under 17 USC 105 and is also made available for use under a CC0 license.

Figure J12. Comparison of the thalamus BSAGE between Juvenile GM1 patients, late-infantile GM1 patients, and neurotypical controls (NC). Late-infantile GM1 patients had an average BSAGE = 38.41, juvenile GM1 patients had an average BSAGE = 22.49, and NC participants had an average BSAGE = -0.24. An analysis of variance (ANOVA) showed statistically significant (F(2, 595) = 558.1, $p < 0.0001$) differences in BSAGE between the cohorts. A post-hoc Tukey test showed the late-infantile cohort had a statistically significantly higher BSAGE compared to both the neurotypical controls ($p < 0.0001$) and juvenile GM1 patients ($p < 0.0001$). The juvenile GM1 cohort also had a significantly higher BSAGE compared to the neurotypical controls ($p < 0.0001$).

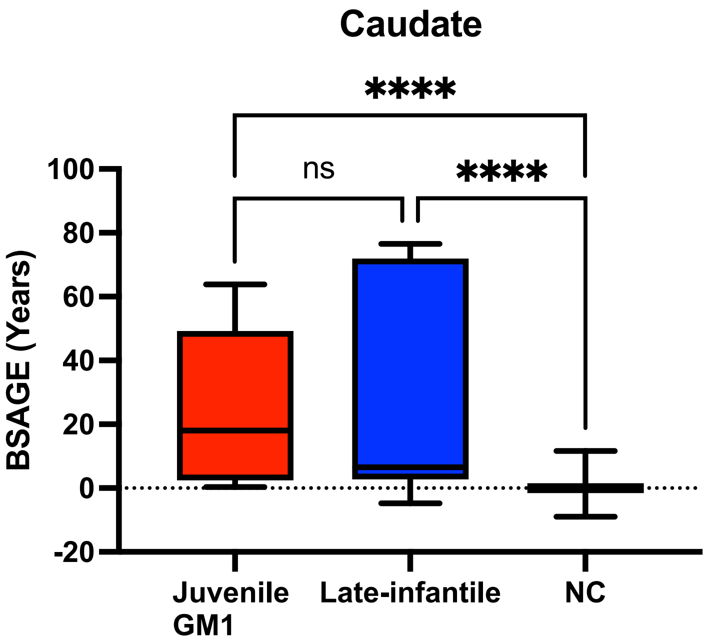

Figure J13. Comparison of the Caudate Nucleus BSAGE between Juvenile GM1 patients, late-infantile GM1 patients, and neurotypical controls (NC). Late-infantile GM1 patients had an average BSAGE = 30.64, juvenile GM1 patients had an average BSAGE = 25.34, and NC participants had an average BSAGE = -0.11. An analysis of variance (ANOVA) showed statistically significant (F(2, 595) = 289.0, $p < 0.0001$) differences in BSAGE between the cohorts. A post-hoc Tukey test showed the late-infantile cohort had a statistically significantly higher BSAGE compared to both the neurotypical controls ($p < 0.0001$) and juvenile GM1 patients ($p < 0.0001$). The juvenile GM1 cohort also had a significantly higher BSAGE compared to the neurotypical controls ($p < 0.0001$).

medRxiv preprint doi: https://doi.org/10.1101/2025.04.23.25326206; this version posted April 25, 2025. The copyright holder for this preprint
(which was not certified by peer review) is the author/funder, who has granted medRxiv a license to display the preprint in perpetuity.
This article is a US Government work. It is not subject to copyright under 17 USC 105 and is also made available
for use under a CC0 license.

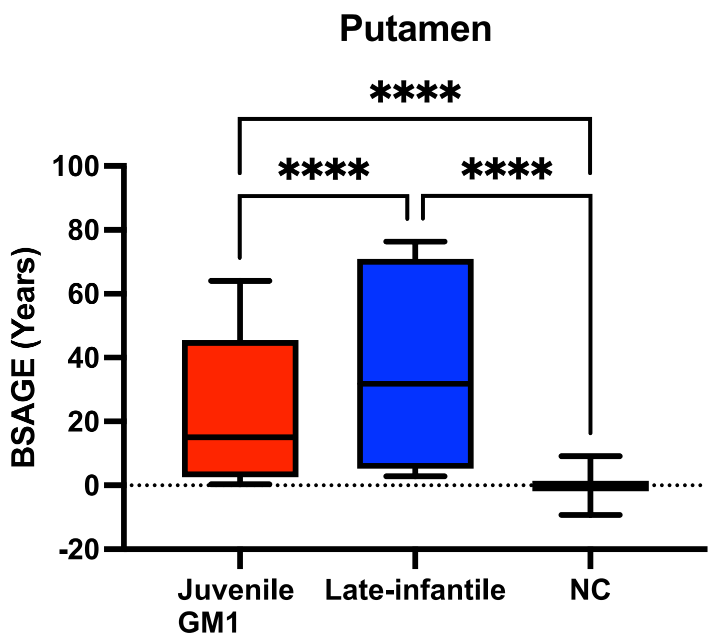

Figure J14. Comparison of the Putamen BSAGE between Juvenile GM1 patients, late-infantile GM1 patients, and neurotypical controls (NC). Late-infantile GM1 patients had an average BSAGE = 38.20, juvenile GM1 patients had an average BSAGE = 25.05, and NC participants had an average BSAGE = -0.19. An analysis of variance (ANOVA) showed statistically significant ($F(2, 595) = 660.8$, $p < 0.0001$) differences in BSAGE between the cohorts. A post-hoc Tukey test showed the late-infantile cohort had a statistically significantly higher BSAGE compared to both the neurotypical controls ($p < 0.0001$) and juvenile GM1 patients ($p < 0.0001$). The juvenile GM1 cohort also had a significantly higher BSAGE compared to the neurotypical controls ($p < 0.0001$).

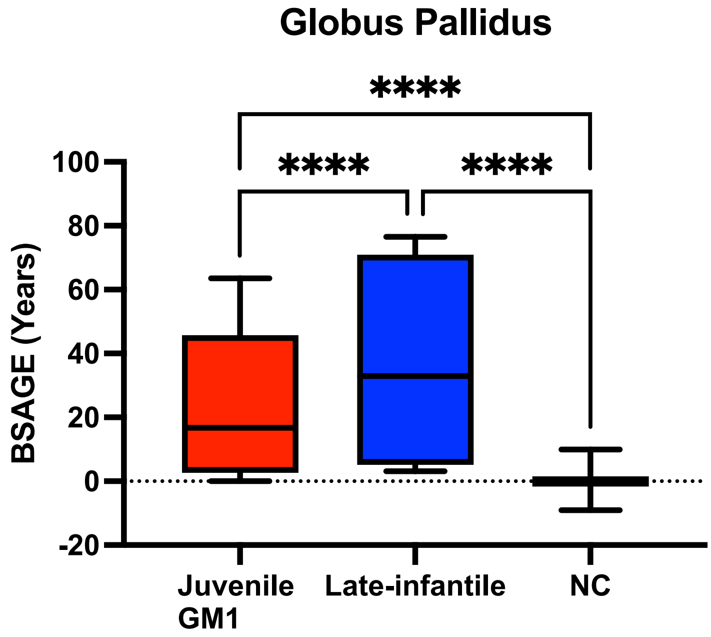

medRxiv preprint doi: https://doi.org/10.1101/2025.04.23.25326206; this version posted April 25, 2025. The copyright holder for this preprint (which was not certified by peer review) is the author/funder, who has granted medRxiv a license to display the preprint in perpetuity. This article is a US Government work. It is not subject to copyright under 17 USC 105 and is also made available for use under a CC0 license.

Figure J15. Comparison of the Globus Pallidus BSAGE between Juvenile GM1 patients, late-infantile GM1 patients, and neurotypical controls (NC). Late-infantile GM1 patients had an average BSAGE = 37.82, juvenile GM1 patients had an average BSAGE = 24.44, and NC participants had an average BSAGE = -0.10. An analysis of variance (ANOVA) showed statistically significant ($F(2, 595) = 438.0$, $p < 0.0001$) differences in BSAGE between the cohorts. A post-hoc Tukey test showed the late-infantile cohort had a statistically significantly higher BSAGE compared to both the neurotypical controls ($p < 0.0001$) and juvenile GM1 patients ($p < 0.0001$). The juvenile GM1 cohort also had a significantly higher BSAGE compared to the neurotypical controls ($p < 0.0001$).

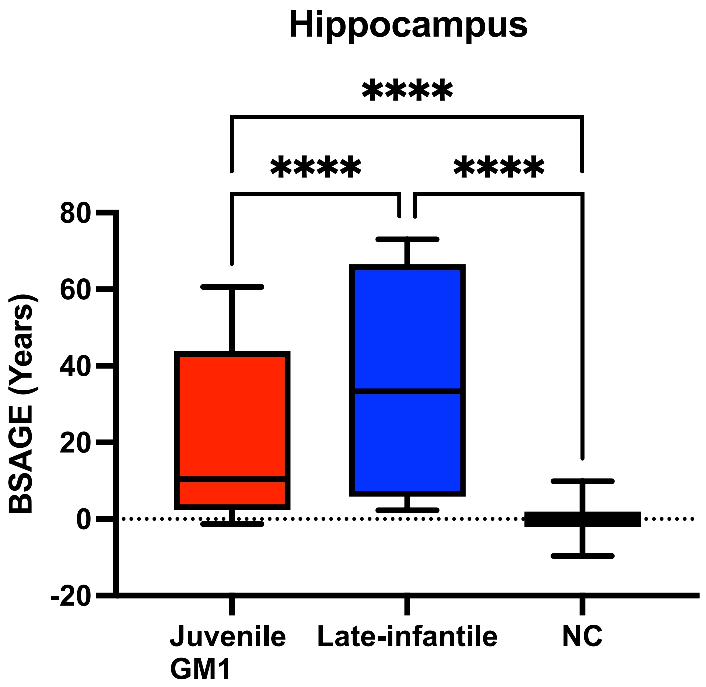

Figure J16. Comparison of the hippocampus BSAGE between Juvenile GM1 patients, late-infantile GM1 patients, and neurotypical controls (NC). Late-infantile GM1 patients had an average BSAGE = 38.10, juvenile GM1 patients had an average BSAGE = 21.87, and NC participants had an average BSAGE = 0.01. An analysis of variance (ANOVA) showed statistically significant ($F(2, 595) = 479.9$, $p < 0.0001$) differences in BSAGE between the cohorts. A post-hoc Tukey test showed the late-infantile cohort had a statistically significantly higher BSAGE compared to both the neurotypical controls ($p < 0.0001$) and juvenile GM1 patients ($p < 0.0001$). The juvenile GM1 cohort also had a significantly higher BSAGE compared to the neurotypical controls ($p < 0.0001$).

medRxiv preprint doi: https://doi.org/10.1101/2025.04.23.25326206; this version posted April 25, 2025. The copyright holder for this preprint (which was not certified by peer review) is the author/funder, who has granted medRxiv a license to display the preprint in perpetuity. This article is a US Government work. It is not subject to copyright under 17 USC 105 and is also made available for use under a CC0 license.

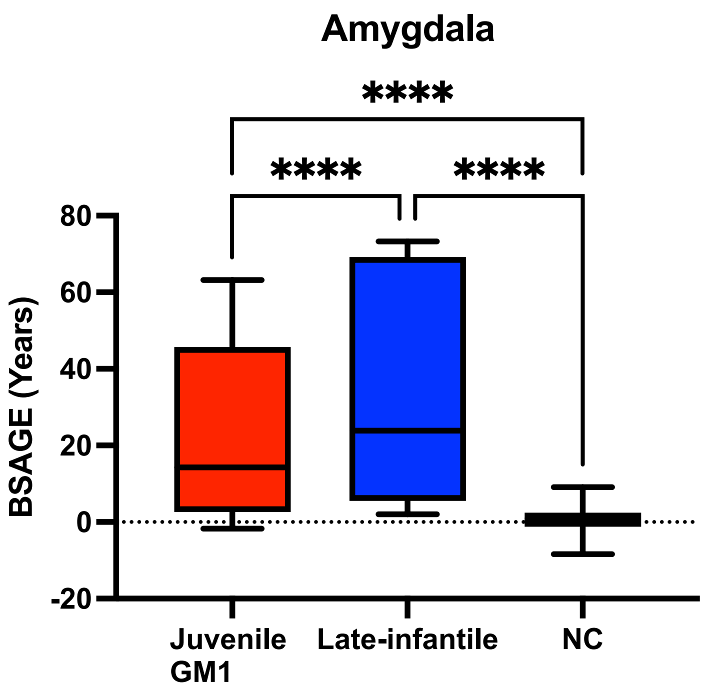

Figure J17. Comparison of the amygdala BSAGE between Juvenile GM1 patients, late-infantile GM1 patients, and neurotypical controls (NC). Late-infantile GM1 patients had an average BSAGE = 36.78, juvenile GM1 patients had an average BSAGE = 23.57, and NC participants had an average BSAGE = 0.55. An analysis of variance (ANOVA) showed statistically significant ($F(2, 595) = 514.7$, $p < 0.0001$) differences in BSAGE between the cohorts. A post-hoc Tukey test showed the late-infantile cohort had a statistically significantly higher BSAGE compared to both the neurotypical controls ($p < 0.0001$) and juvenile GM1 patients ($p < 0.0001$). The juvenile GM1 cohort also had a significantly higher BSAGE compared to the neurotypical controls ($p < 0.0001$).

medRxiv preprint doi: https://doi.org/10.1101/2025.04.23.25326206; this version posted April 25, 2025. The copyright holder for this preprint (which was not certified by peer review) is the author/funder, who has granted medRxiv a license to display the preprint in perpetuity.
This article is a US Government work. It is not subject to copyright under 17 USC 105 and is also made available for use under a CC0 license.

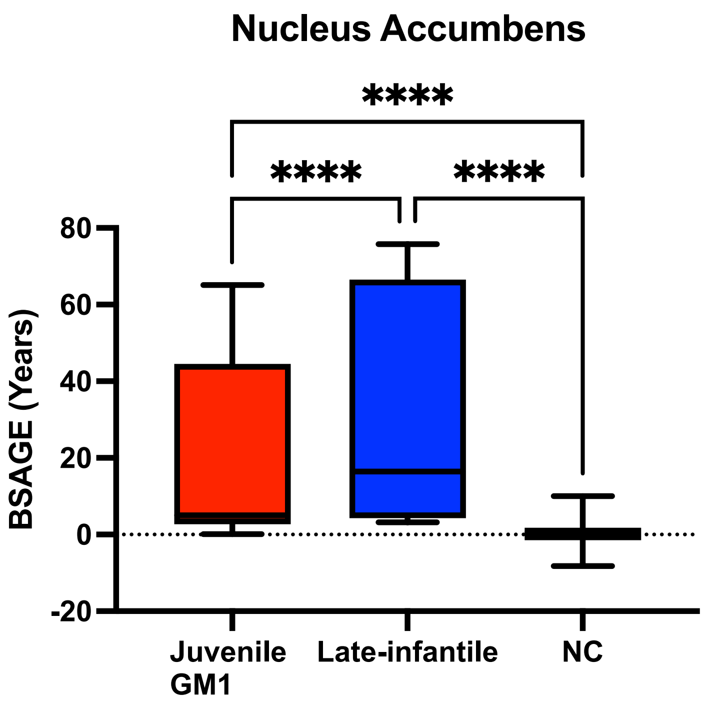

Figure J18. Comparison of the nucleus accumbens (NAC) BSAGE between Juvenile GM1 patients, late-infantile GM1 patients, and neurotypical controls (NC). Late-infantile GM1 patients had an average BSAGE = 30.56, juvenile GM1 patients had an average BSAGE = 23.46, and NC participants had an average BSAGE = 0.18. An analysis of variance (ANOVA) showed statistically significant ($F(2, 595) = 373.2$, $p < 0.0001$) differences in BSAGE between the cohorts. A post-hoc Tukey test showed the late-infantile cohort had a statistically significantly higher BSAGE compared to both the neurotypical controls ($p < 0.0001$) and juvenile GM1 patients ($p < 0.0001$). The juvenile GM1 cohort also had a significantly higher BSAGE compared to the neurotypical controls ($p < 0.0001$).

medRxiv preprint doi: https://doi.org/10.1101/2025.04.23.25326206; this version posted April 25, 2025. The copyright holder for this preprint
(which was not certified by peer review) is the author/funder, who has granted medRxiv a license to display the preprint in perpetuity.
This article is a US Government work. It is not subject to copyright under 17 USC 105 and is also made available
for use under a CC0 license.

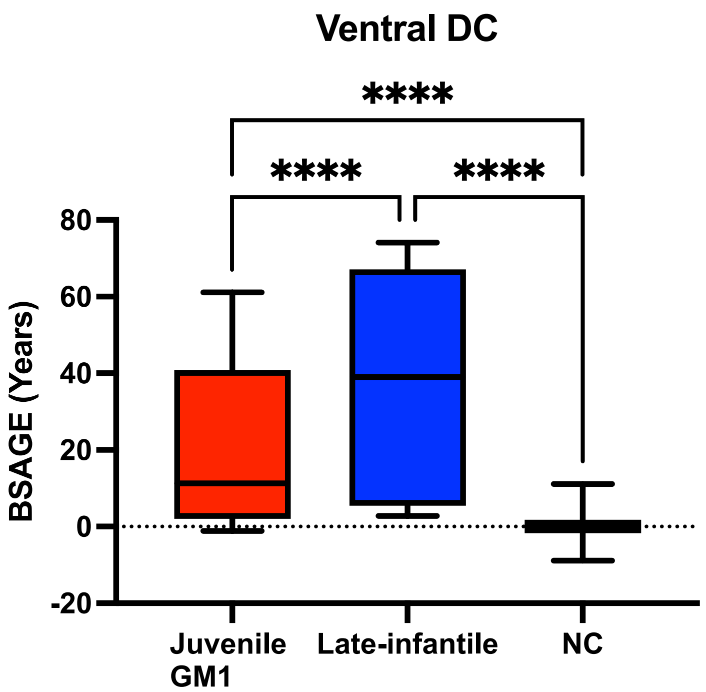

Figure J19. Comparison of the ventral diencephalon BSAGE between Juvenile GM1 patients, late-infantile GM1 patients, and neurotypical controls (NC). Late-infantile GM1 patients had an average BSAGE = 39.12, juvenile GM1 patients had an average BSAGE = 21.10, and NC participants had an average BSAGE = 0.08. An analysis of variance (ANOVA) showed statistically significant ($F(2, 595) = 532.3$, $p < 0.0001$) differences in BSAGE between the cohorts. A post-hoc Tukey test showed the late-infantile cohort had a statistically significantly higher BSAGE compared to both the neurotypical controls ($p < 0.0001$) and juvenile GM1 patients ($p < 0.0001$). The juvenile GM1 cohort also had a significantly higher BSAGE compared to the neurotypical controls ($p < 0.0001$).

medRxiv preprint doi: https://doi.org/10.1101/2025.04.23.25326206; this version posted April 25, 2025. The copyright holder for this preprint (which was not certified by peer review) is the author/funder, who has granted medRxiv a license to display the preprint in perpetuity.
This article is a US Government work. It is not subject to copyright under 17 USC 105 and is also made available for use under a CC0 license.

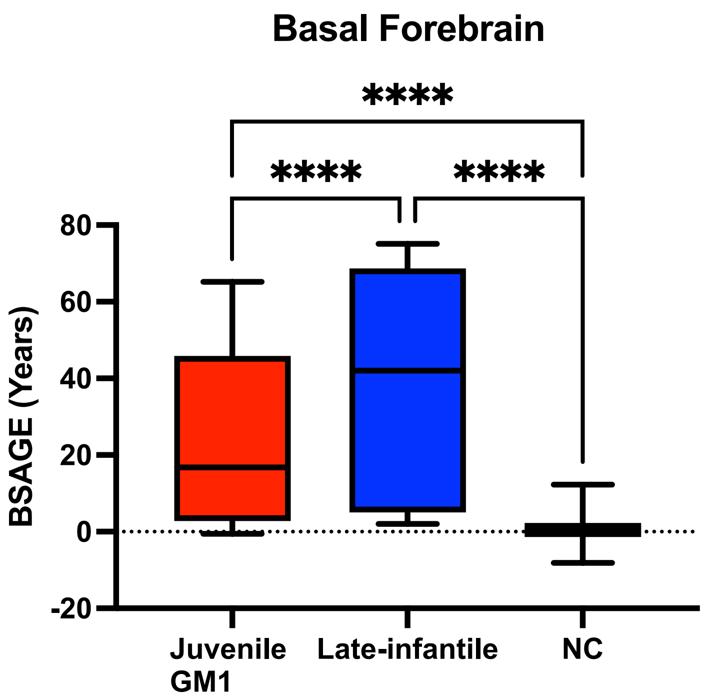

Figure J20. Comparison of the basal forebrain BSAGE between Juvenile GM1 patients, late-infantile GM1 patients, and neurotypical controls (NC). Late-infantile GM1 patients had an average BSAGE = 39.00, juvenile GM1 patients had an average BSAGE = 24.66, and NC participants had an average BSAGE = 0.45. An analysis of variance (ANOVA) showed statistically significant ($F(2, 595) = 717.8$, $p < 0.0001$) differences in BSAGE between the cohorts. A post-hoc Tukey test showed the late-infantile cohort had a statistically significantly higher BSAGE compared to both the neurotypical controls ($p < 0.0001$) and juvenile GM1 patients ($p < 0.0001$). The juvenile GM1 cohort also had a significantly higher BSAGE compared to the neurotypical controls ($p < 0.0001$).

medRxiv preprint doi: https://doi.org/10.1101/2025.04.23.25326206; this version posted April 25, 2025. The copyright holder for this preprint (which was not certified by peer review) is the author/funder, who has granted medRxiv a license to display the preprint in perpetuity. This article is a US Government work. It is not subject to copyright under 17 USC 105 and is also made available for use under a CC0 license.

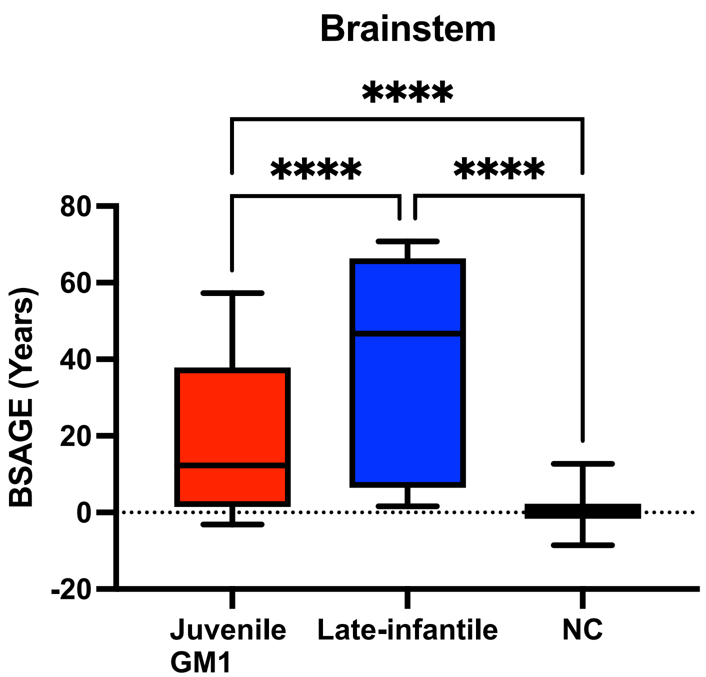

Figure J21. Comparison of the brainstem BSAGE between Juvenile GM1 patients, late-infantile GM1 patients, and neurotypical controls (NC). Late-infantile GM1 patients had an average BSAGE = 38.56, juvenile GM1 patients had an average BSAGE = 19.56, and NC participants had an average BSAGE = 0.40. An analysis of variance (ANOVA) showed statistically significant ($F(2, 595) = 429.3$, $p < 0.0001$) differences in BSAGE between the cohorts. A post-hoc Tukey test showed the late-infantile cohort had a statistically significantly higher BSAGE compared to both the neurotypical controls ($p < 0.0001$) and juvenile GM1 patients ($p < 0.0001$). The juvenile GM1 cohort also had a significantly higher BSAGE compared to the neurotypical controls ($p < 0.0001$).

medRxiv preprint doi: https://doi.org/10.1101/2025.04.23.25326206; this version posted April 25, 2025. The copyright holder for this preprint (which was not certified by peer review) is the author/funder, who has granted medRxiv a license to display the preprint in perpetuity. This article is a US Government work. It is not subject to copyright under 17 USC 105 and is also made available for use under a CC0 license.

Supplement K: Comparisons Between Predicted Brain Age and Brain Volumetrics

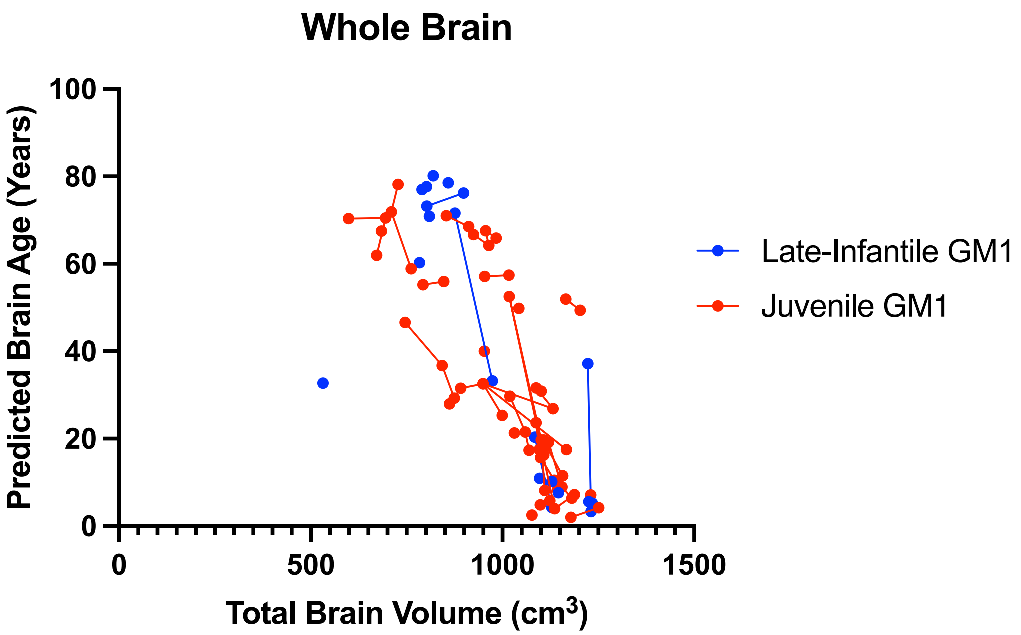

Figure K1. Correlation between whole brain volume (cm^3) and predicted whole brain age in juvenile and late-infantile GM1 patients. Total brain volume correlated with predicted whole brain age in both the juvenile ($R^2 = 0.57$) and late-infantile ($R^2 = 0.46$) patients.

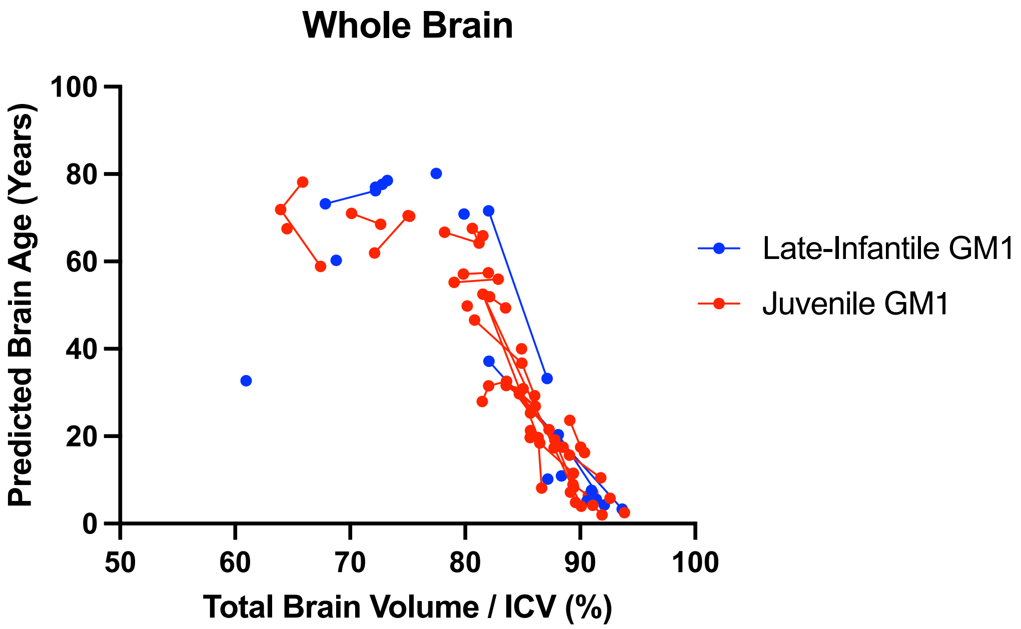

Figure K2. Correlation between whole brain volume controlled for total intracranial volume (ICV) and predicted whole brain age in juvenile and late-infantile GM1 patients. Total brain

medRxiv preprint doi: https://doi.org/10.1101/2025.04.23.25326206; this version posted April 25, 2025. The copyright holder for this preprint (which was not certified by peer review) is the author/funder, who has granted medRxiv a license to display the preprint in perpetuity. This article is a US Government work. It is not subject to copyright under 17 USC 105 and is also made available for use under a CC0 license.

volume correlated with predicted whole brain age in both the juvenile ($R^2 = 0.77$) and late-infantile ($R^2 = 0.51$) patients.

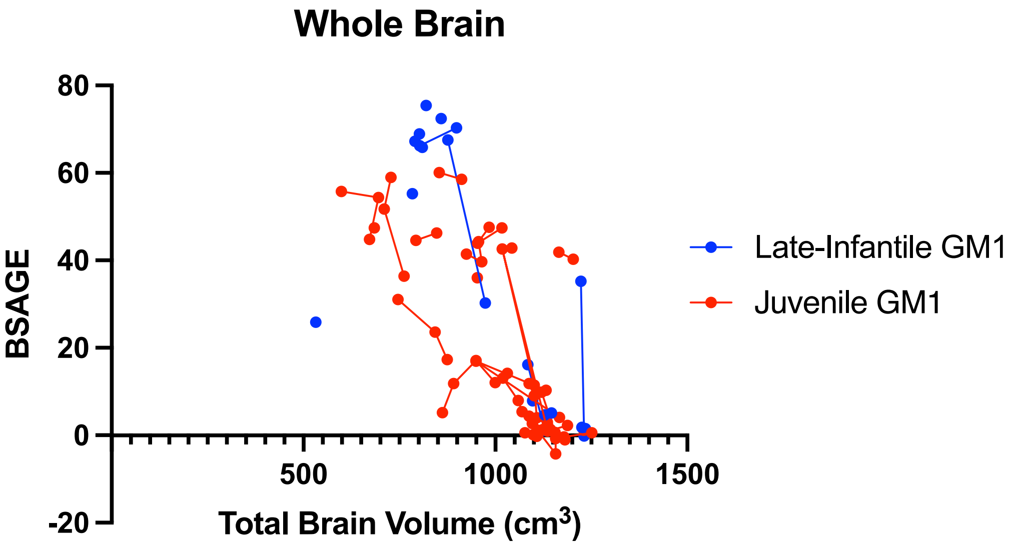

Figure K3. Correlation between whole brain volume (cm^3) and predicted whole brain, brain structures age gap estimation (BSAGE) in juvenile and late-infantile GM1 patients. Total brain volume correlated with predicted whole brain age in both the juvenile ($R^2 = 0.49$) and late-infantile ($R^2 = 0.43$) patients.

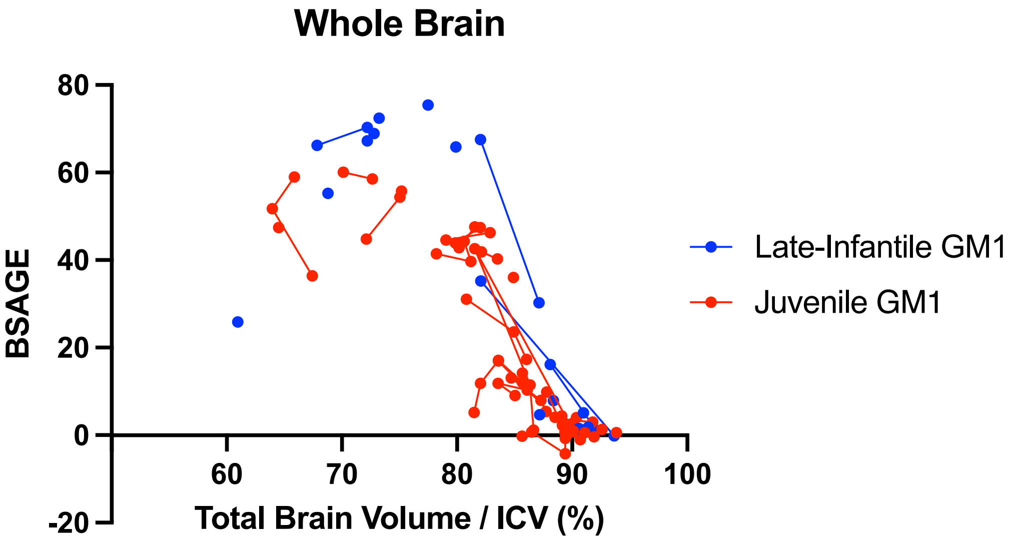

Figure K4. Correlation between whole brain volume controlled for total intracranial volume (ICV) and predicted whole brain, brain structures age gap estimation (BSAGE) in juvenile and late-infantile GM1 patients. Total brain volume correlated with predicted whole brain age in both the juvenile ($R^2 = 0.69$) and late-infantile ($R^2 = 0.47$) patients.

medRxiv preprint doi: https://doi.org/10.1101/2025.04.23.25326206; this version posted April 25, 2025. The copyright holder for this preprint (which was not certified by peer review) is the author/funder, who has granted medRxiv a license to display the preprint in perpetuity.
This article is a US Government work. It is not subject to copyright under 17 USC 105 and is also made available for use under a CC0 license.

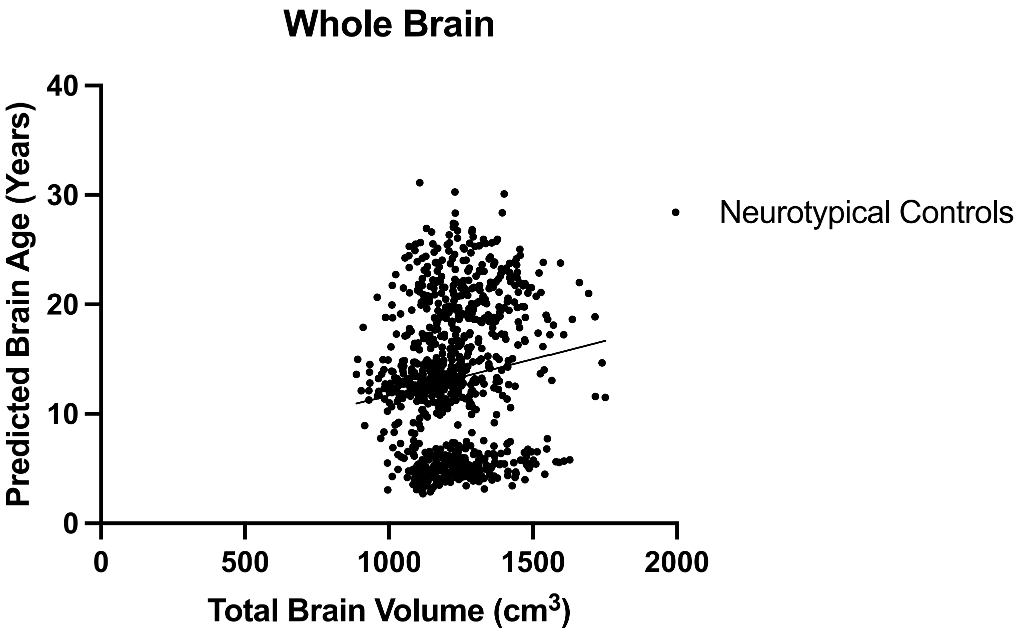

Figure K5. Correlation between whole brain volume (cm^3) and predicted whole brain age in neurotypical controls. Total brain volume correlated with predicted whole brain age in the neurotypical controls ($R^2 = 0.02$).

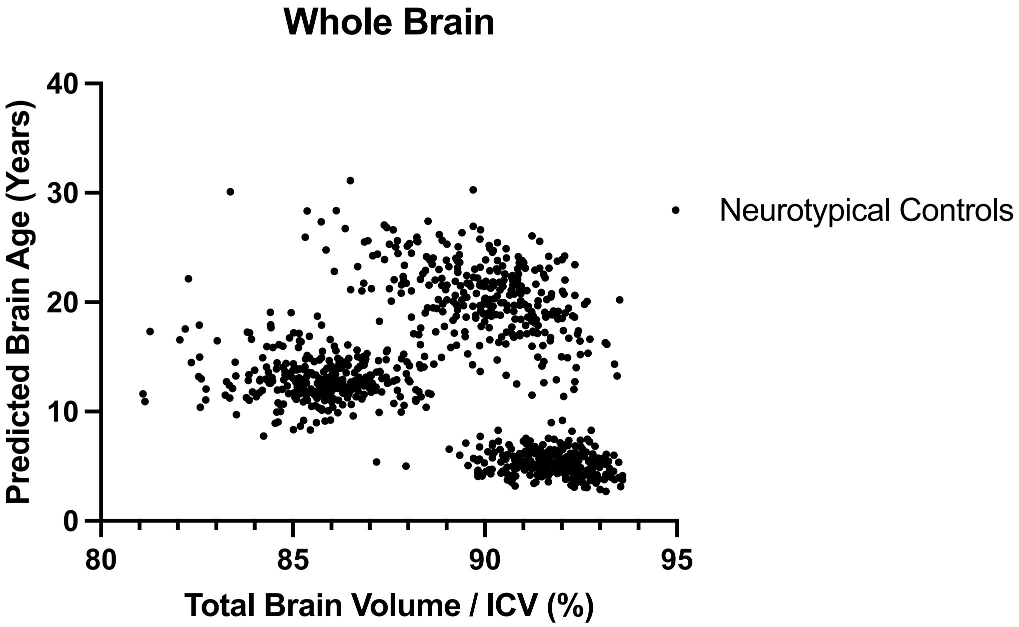

Figure K6. Correlation between whole brain volume controlled for total intracranial volume (ICV) and predicted whole brain age in neurotypical controls. Total brain volume correlated with predicted whole brain age in the neurotypical controls ($R^2 = 0.07$).

medRxiv preprint doi: https://doi.org/10.1101/2025.04.23.25326206; this version posted April 25, 2025. The copyright holder for this preprint (which was not certified by peer review) is the author/funder, who has granted medRxiv a license to display the preprint in perpetuity.
This article is a US Government work. It is not subject to copyright under 17 USC 105 and is also made available for use under a CC0 license.

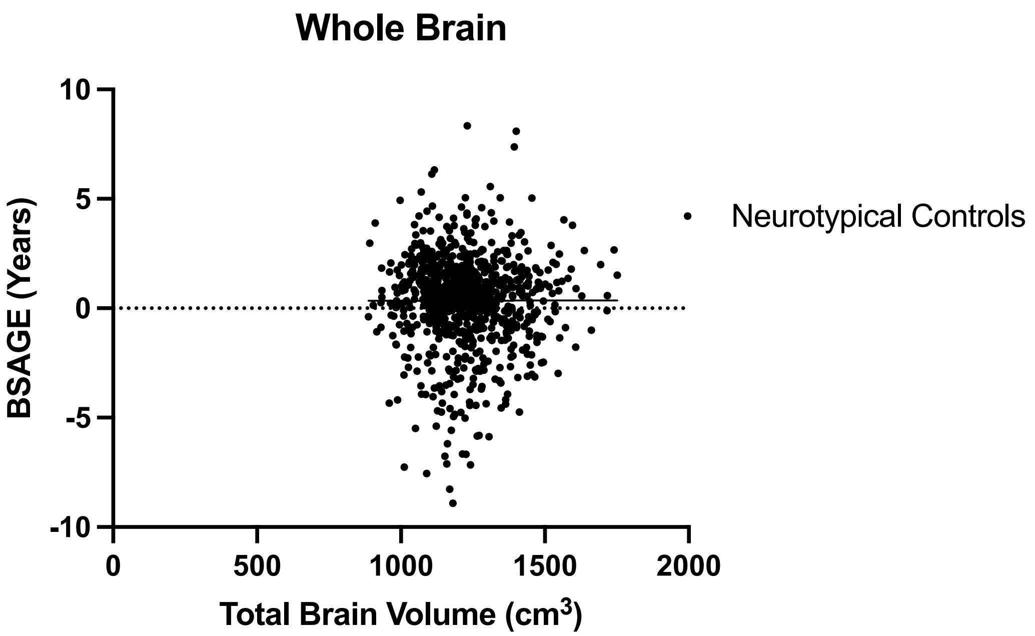

Figure K7. Correlation between whole brain volume (cm^3) and predicted whole brain, brain structures age gap estimation (BSAGE) in neurotypical controls. Total brain volume did not correlate with predicted whole brain age in the neurotypical controls ($R^2 < 0.01$).

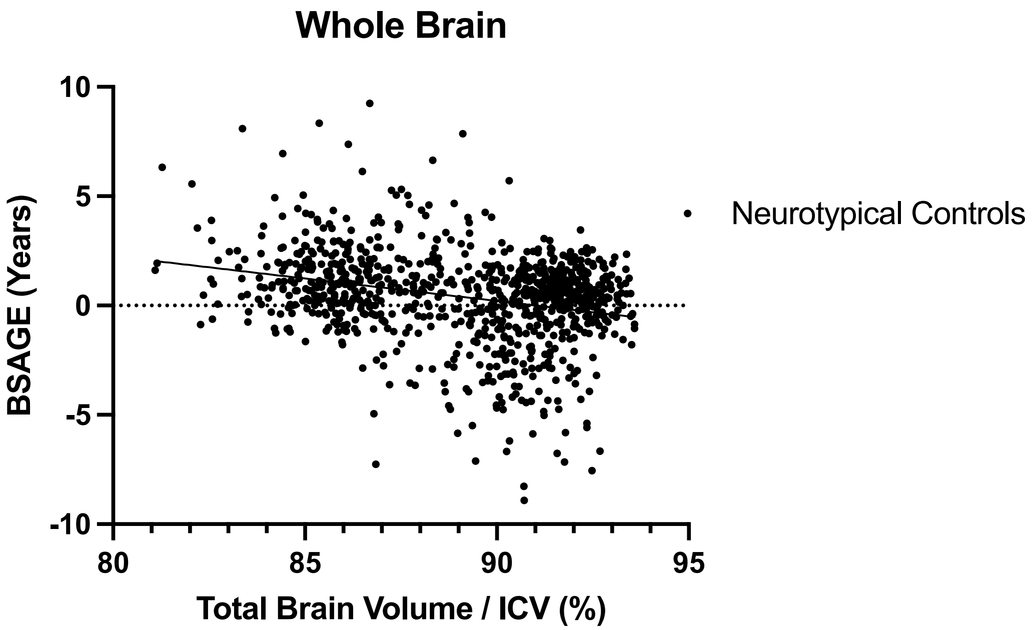

Figure K8. Correlation between whole brain volume controlled for total intracranial volume (ICV) and predicted whole brain, brain structures age gap estimation (BSAGE) in neurotypical controls. Total brain volume correlated with predicted whole brain age in the neurotypical controls ($R^2 = 0.08$).

medRxiv preprint doi: https://doi.org/10.1101/2025.04.23.25326206; this version posted April 25, 2025. The copyright holder for this preprint (which was not certified by peer review) is the author/funder, who has granted medRxiv a license to display the preprint in perpetuity.
This article is a US Government work. It is not subject to copyright under 17 USC 105 and is also made available for use under a CC0 license.

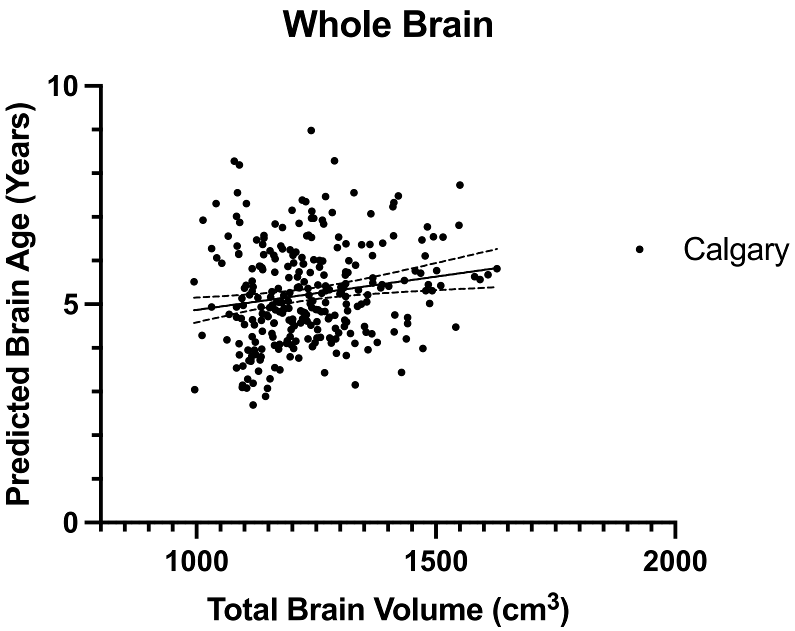

Figure K9. Correlation between whole brain volume (cm^3) and predicted whole brain age in Calgary neurotypical controls. Total brain volume did correlated with predicted whole brain age in Calgary neurotypical controls (R^2 = 0.03).

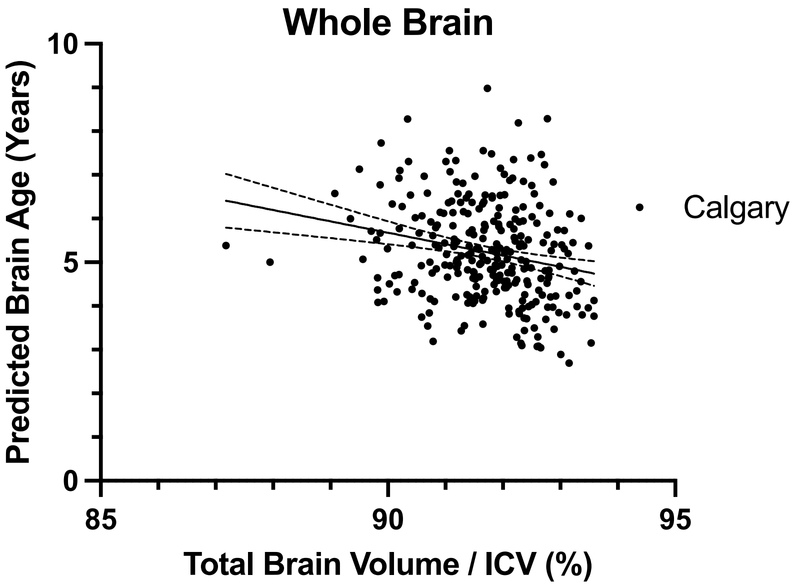

Figure K10. Correlation between whole brain volume controlled for total intracranial volume (ICV) and predicted whole brain, brain structures age gap estimation (BSAGE) in Calgary neurotypical controls. Total brain volume correlated with predicted whole brain age in the Calgary neurotypical controls (R^2 = 0.05).

medRxiv preprint doi: https://doi.org/10.1101/2025.04.23.25326206; this version posted April 25, 2025. The copyright holder for this preprint (which was not certified by peer review) is the author/funder, who has granted medRxiv a license to display the preprint in perpetuity. This article is a US Government work. It is not subject to copyright under 17 USC 105 and is also made available for use under a CC0 license.

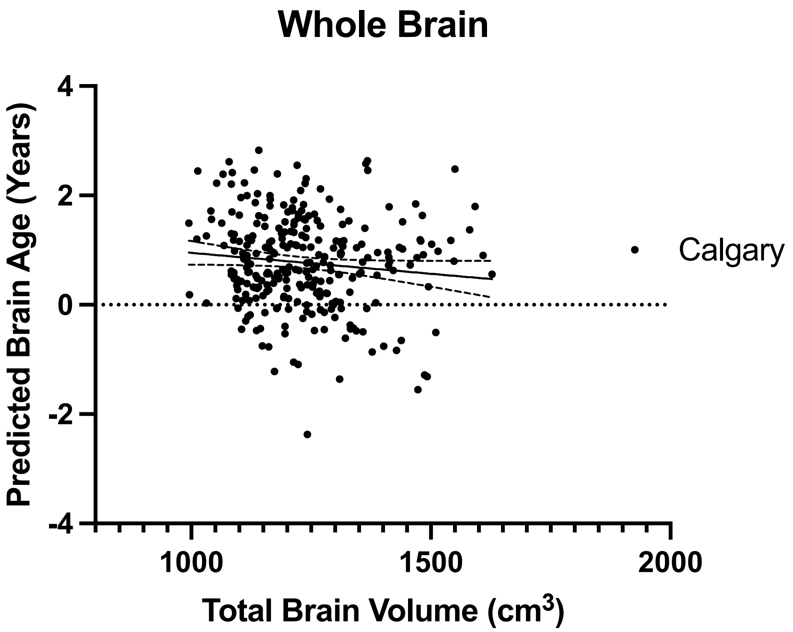

Figure K11. Correlation between whole brain volume (cm^3) and predicted whole brain, brain structures age gap estimation (BSAGE) in neurotypical controls. Total brain volume did not correlate with predicted whole brain age in the neurotypical controls ($R^2 = 0.01$).

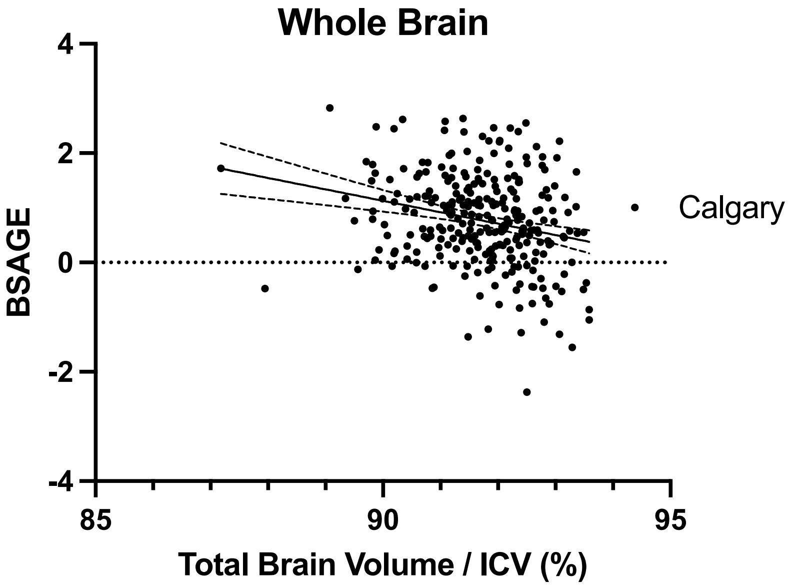

Figure K12. Correlation between whole brain volume controlled for ICV and predicted whole brain, brain structures age gap estimation (BSAGE) in neurotypical controls. Total brain volume did not correlate with predicted whole brain age in the neurotypical controls ($R^2 = 0.06$).

medRxiv preprint doi: https://doi.org/10.1101/2025.04.23.25326206; this version posted April 25, 2025. The copyright holder for this preprint
(which was not certified by peer review) is the author/funder, who has granted medRxiv a license to display the preprint in perpetuity.
This article is a US Government work. It is not subject to copyright under 17 USC 105 and is also made available
for use under a CC0 license.

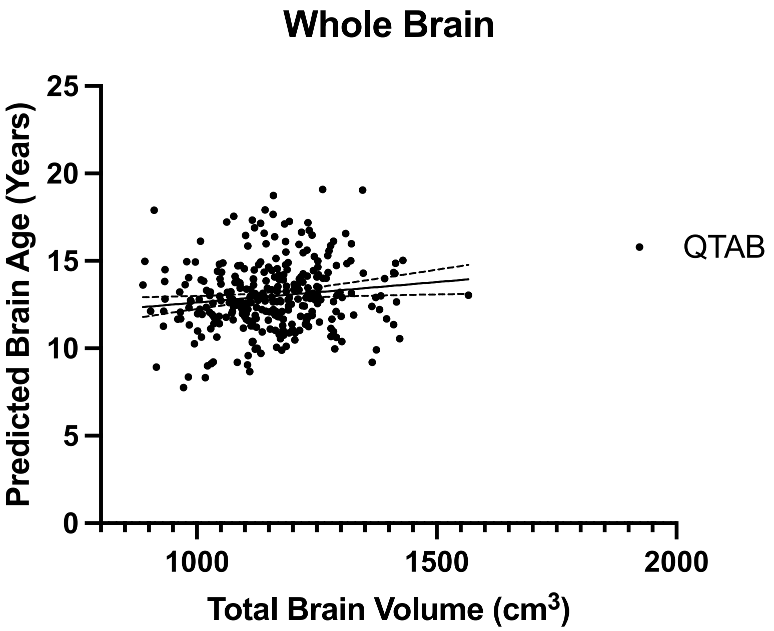

Figure K13. Correlation between whole brain volume and predicted whole brain age gap estimation in QTAB neurotypical controls. Total brain volume correlated with predicted whole brain age in the QTAB neurotypical controls ($R^2 = 0.02$).

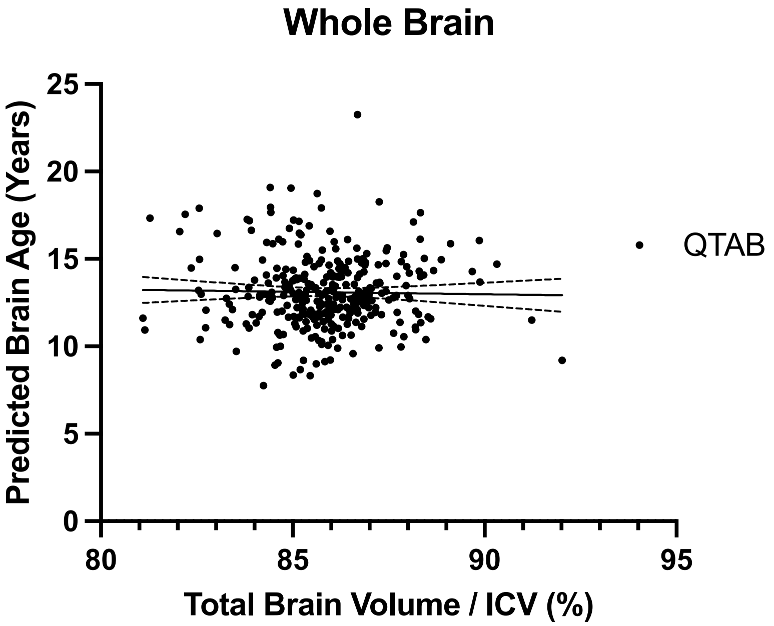

Figure K14. Correlation between whole brain volume controlled for total intracranial volume (ICV) and predicted whole brain age gap estimation in QTAB neurotypical controls. Total brain volume correlated with predicted whole brain age in the QTAB neurotypical controls ($R^2 < 0.01$).

medRxiv preprint doi: https://doi.org/10.1101/2025.04.23.25326206; this version posted April 25, 2025. The copyright holder for this preprint (which was not certified by peer review) is the author/funder, who has granted medRxiv a license to display the preprint in perpetuity.
This article is a US Government work. It is not subject to copyright under 17 USC 105 and is also made available for use under a CC0 license.

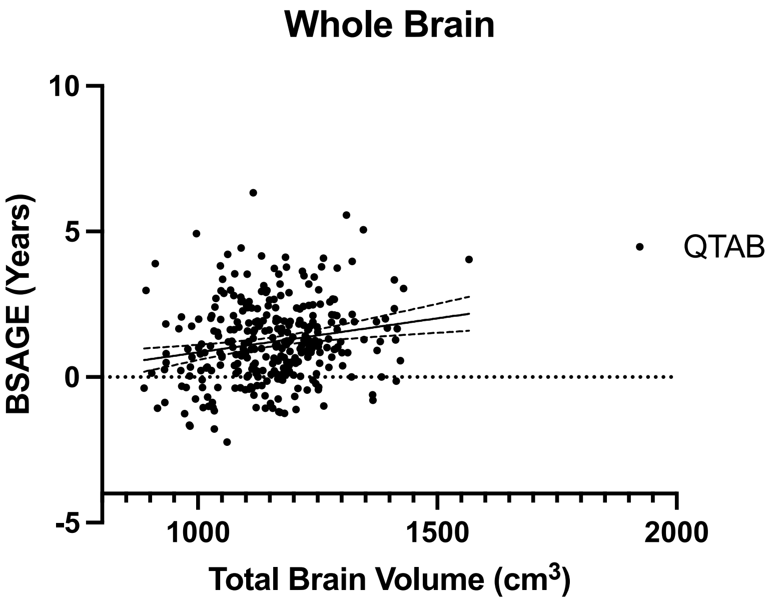

Figure K15. Correlation between whole brain volume (cm^3) and predicted whole brain, brain structures age gap estimation (BSAGE) in the QTAB neurotypical controls. Total brain volume correlated with predicted whole brain age in the QTAB neurotypical controls ($R^2 = 0.04$).

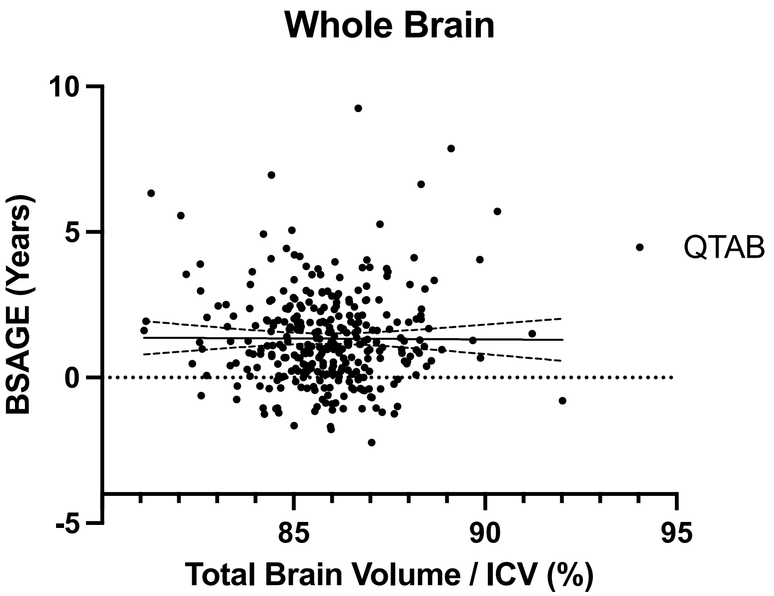

Figure K16. Correlation between whole brain volume controlled for total intracranial volume (ICV) and predicted whole brain, brain structures age gap estimation (BSAGE) in the QTAB neurotypical controls. Total brain volume did not correlate with predicted whole brain age in the QTAB neurotypical controls ($R^2 < 0.01$).

medRxiv preprint doi: https://doi.org/10.1101/2025.04.23.25326206; this version posted April 25, 2025. The copyright holder for this preprint
(which was not certified by peer review) is the author/funder, who has granted medRxiv a license to display the preprint in perpetuity.
This article is a US Government work. It is not subject to copyright under 17 USC 105 and is also made available
for use under a CC0 license.

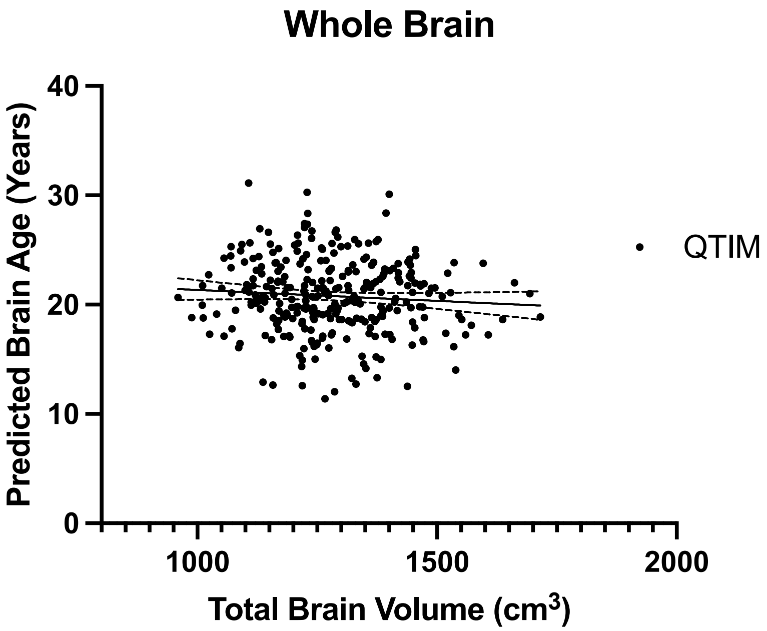

Figure K17. Correlation between whole brain volume and predicted whole brain age gap estimation in QTIM neurotypical controls. Total brain volume correlated with predicted whole brain age in the QTIM neurotypical controls ($R^2 = 0.01$).

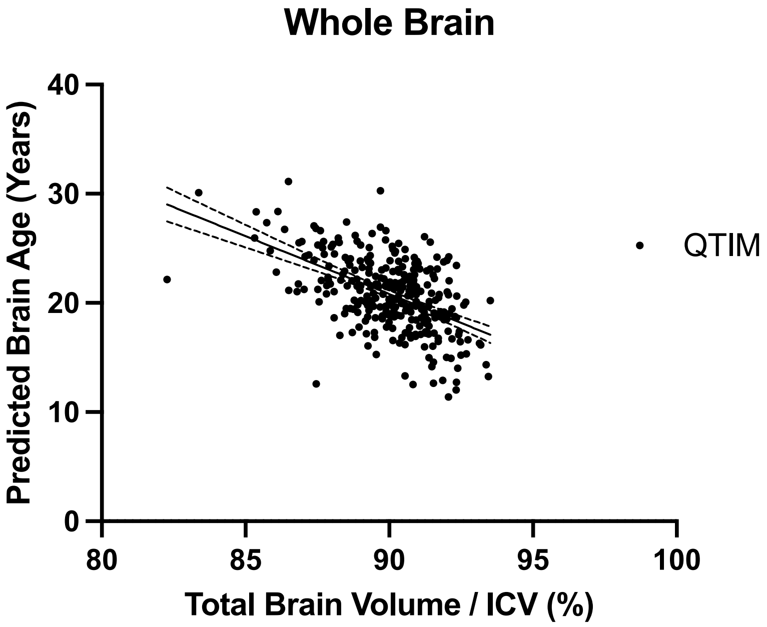

Figure K18. Correlation between whole brain volume controlled for ICV and predicted whole brain age gap estimation in QTIM neurotypical controls. Total brain volume correlated with predicted whole brain age in the QTIM neurotypical controls ($R^2 = 0.28$).

medRxiv preprint doi: https://doi.org/10.1101/2025.04.23.25326206; this version posted April 25, 2025. The copyright holder for this preprint
(which was not certified by peer review) is the author/funder, who has granted medRxiv a license to display the preprint in perpetuity.
This article is a US Government work. It is not subject to copyright under 17 USC 105 and is also made available
for use under a CC0 license.

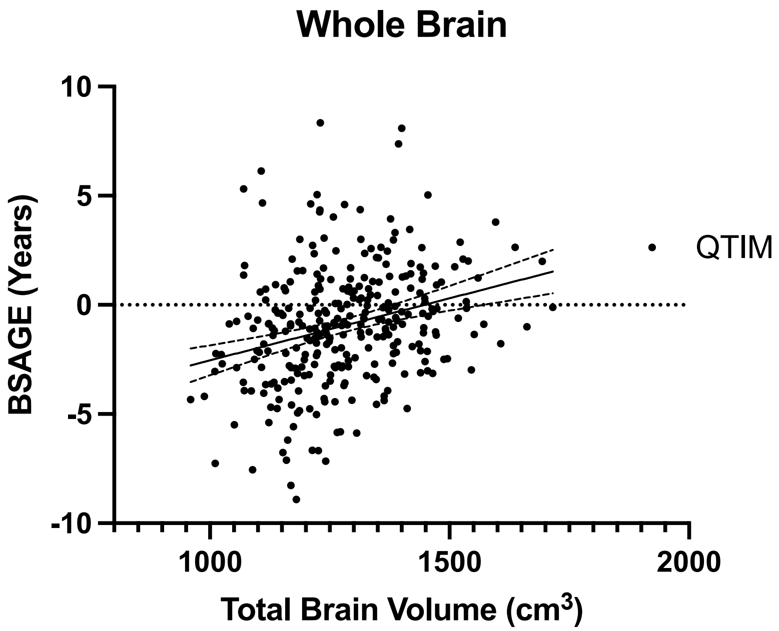

Figure K19. Correlation between whole brain volume (cm^3) and predicted whole brain, brain structures age gap estimation (BSAGE) in the QTIM neurotypical controls. Total brain volume correlated with predicted whole brain age in the QTIM neurotypical controls ($R^2 = 0.08$).

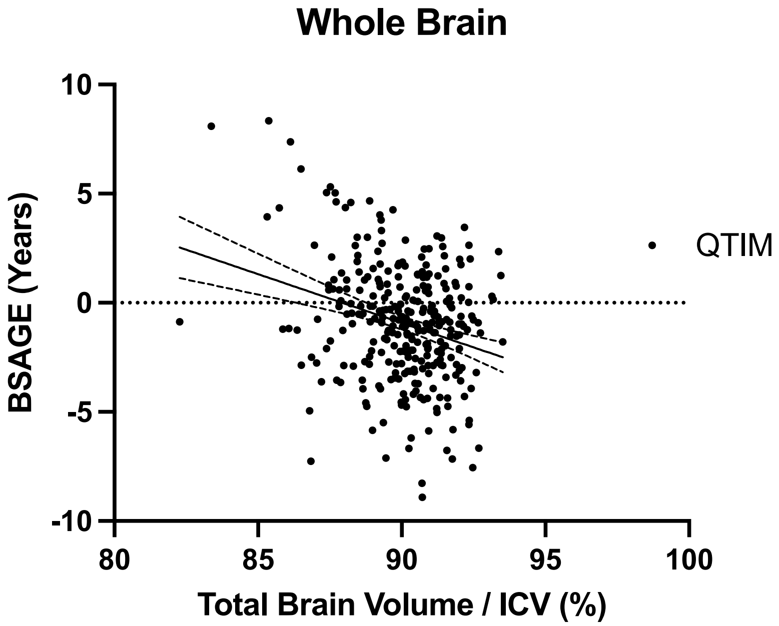

Figure K20. Correlation between whole brain volume controlled for total intracranial volume (ICV) and predicted whole brain, brain structures age gap estimation (BSAGE) in the QTIM neurotypical controls. Total brain volume correlated with predicted whole brain BSAGE in the QTIM neurotypical controls ($R^2 = 0.08$).

medRxiv preprint doi: https://doi.org/10.1101/2025.04.23.25326206; this version posted April 25, 2025. The copyright holder for this preprint (which was not certified by peer review) is the author/funder, who has granted medRxiv a license to display the preprint in perpetuity. This article is a US Government work. It is not subject to copyright under 17 USC 105 and is also made available for use under a CC0 license.

Supplementary References

1. National Human Genome Research Institute. Natural History of Glycosphingolipid Storage Disorders and Glycoprotein Disorders ClinicalTrials.gov identifier: NCT00029965. Updated August 7, 2024. Accessed August 12, 2024. https://clinicaltrials.gov/study/NCT00029965.
2. Reynolds JE, Long X, Paniukov D, Bagshawe M, Lebel C. Calgary Preschool magnetic resonance imaging (MRI) dataset. Data Brief. 2020;29:105224. Published 2020 Jan 31. doi:10.1016/j.dib.2020.105224
3. Reynolds, J., Long, X., Paniukov, D., Bagshawe, M., Dewey, D., & Lebel, C. (2023, June 13). Calgary Preschool MRI Dataset. https://doi.org/10.17605/OSF.IO/AXZ5R.
4. Strike, Lachlan T. and Hansell, Narelle K. and Miller, Jessica L. and Chuang, Kai-Hsiang and Thompson, Paul M. and de Zubicaray, Greig I. and McMahon, Katie L. and Wright, Margaret J. (2022). Queensland Twin Adolescent Brain (QTAB). OpenNeuro. [Dataset] doi: doi:10.18112/openneuro.ds004146.v1.0.3.
5. Strike, L.T., Hansell, N.K., Chuang, KH. *et al.* The Queensland Twin Adolescent Brain Project, a longitudinal study of adolescent brain development. *Sci Data* **10**, 195 (2023). https://doi.org/10.1038/s41597-023-02038-w.
6. Strike L. T., Hansell N. K., Couvy-Duchesne B., Thompson P. M., de Zubicaray G. I., McMahon K. L., Wright M. J. Genetic Complexity of Cortical Structure: Differences in Genetic and Environmental Factors Influencing Cortical Surface Area and Thickness. Cereb Cortex. 2019 Mar 1;29(3):952-962. https://doi.org/10.1093/cercor/bhy002.
7. Strike, Lachlan T. and Blokland, Gabriella A.M. and Hansell, Narelle K. and Martin, Nicholas G. and Toga, Arthur W. and Thompson, Paul M. and de Zubicaray, Greig I. and McMahon, Katie L. and Wright, Margaret J. (2022). Queensland Twin IMaging (QTIM). OpenNeuro. [Dataset] doi: doi:10.18112/openneuro.ds004169.v1.0.6.
8. Dessau RB, Pipper CB. R--en programpakke til statistisk databehandling og grafik ["R"--project for statistical computing]. *Ugeskr Laeger*. 2008;170(5):328-330.
9. Winter B, Linear models and linear mixed effects models in R with linguistic applications", 2013. 10.48550/arXiv.1308.5499.
10. Mittal N, Thakkar B, Hodges CB, et al. Effect of neuroanatomy on corticomotor excitability during and after transcranial magnetic stimulation and intermittent theta burst stimulation. Hum Brain Mapp. 2022;43(14):4492-4507. doi:10.1002/hbm.25968.
11. Lewis CJ, Johnston JM, Zaragoza Domingo S, et al. Retrospective assessment of clinical global impression of severity and change in GM1 gangliosidosis: a tool to score natural history data in rare disease cohorts. *Orphanet J Rare Dis*. 2025;20(1):125. Published 2025 Mar 14. doi:10.1186/s13023-025-03614-6
